# Ubiquitin-Mediated Regulation of RIPK1 Kinase Activity Independent of IKK and MK2

**DOI:** 10.1016/j.molcel.2018.01.027

**Published:** 2018-02-15

**Authors:** Alessandro Annibaldi, Sidonie Wicky John, Tom Vanden Berghe, Kirby N. Swatek, Jianbin Ruan, Gianmaria Liccardi, Katiuscia Bianchi, Paul R. Elliott, Sze Men Choi, Samya Van Coillie, John Bertin, Hao Wu, David Komander, Peter Vandenabeele, John Silke, Pascal Meier

**Affiliations:** 1 The Breast Cancer Now Toby Robins Research Centre, The Institute of Cancer Research, London, UK; 2 VIB Center for Inflammation Research, Ghent, Belgium; 3 Department of Biomedical Molecular Biology, Ghent University, Ghent, Belgium; 4 Medical Research Council, Laboratory of Molecular Biology, Cambridge, UK; 5 Department of Biological Chemistry and Molecular Pharmacology, Harvard Medical School, Room 3024B, 3 Blackfan Circle, Boston, MA 02115, USA; 6 Centre for Molecular Oncology, Barts Cancer Institute, Queen Mary University of London, London, UK; 7 Pattern Recognition Receptor DPU and Platform Technology and Science, GlaxoSmithKline, Collegeville Road, Collegeville, PA 19426, USA; 8 The Walter and Eliza Hall Institute of Medical Research, Parkville, VIC 3052, Australia; 9 Department of Medical Biology, University of Melbourne, Parkville, VIC 3050, Australia

**Keywords:** TNF, cIAPs, ubiquitin, RIPK1, cell death, apoptosis, necroptosis, caspase-8, inflammation

## Abstract

Tumor necrosis factor (TNF) can drive inflammation, cell survival, and death. While ubiquitylation-, phosphorylation-, and nuclear factor κB (NF-κB)-dependent checkpoints suppress the cytotoxic potential of TNF, it remains unclear whether ubiquitylation can directly repress TNF-induced death. Here, we show that ubiquitylation regulates RIPK1’s cytotoxic potential not only via activation of downstream kinases and NF-kB transcriptional responses, but also by directly repressing RIPK1 kinase activity via ubiquitin-dependent inactivation. We find that the ubiquitin-associated (UBA) domain of cellular inhibitor of apoptosis (cIAP)1 is required for optimal ubiquitin-lysine occupancy and K48 ubiquitylation of RIPK1. Independently of IKK and MK2, cIAP1-mediated and UBA-assisted ubiquitylation suppresses RIPK1 kinase auto-activation and, in addition, marks it for proteasomal degradation. In the absence of a functional UBA domain of cIAP1, more active RIPK1 kinase accumulates in response to TNF, causing RIPK1 kinase-mediated cell death and systemic inflammatory response syndrome. These results reveal a direct role for cIAP-mediated ubiquitylation in controlling RIPK1 kinase activity and preventing TNF-mediated cytotoxicity.

## Highlights

• Ubiquitylation directly controls RIPK1 kinase activity in TNF signaling
• UBA-dependent ubiquitylation of RIPK1 represses its kinase activity and cell death
• The UBA contributes to optimal occupancy of ubiquitin-acceptor lysines in RIPK1
• UBA-dependent ubiquitylation of RIPK1 also targets it for proteasomal degradation

## Graphical Abstract

**Figure:**
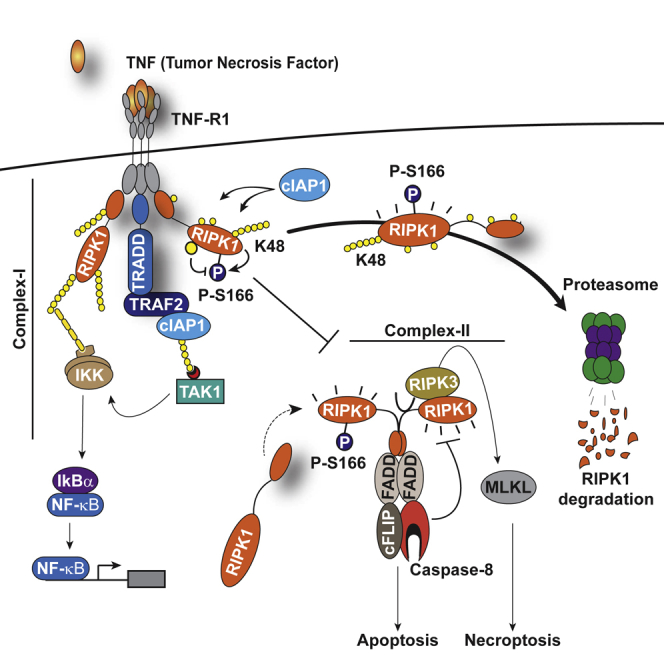

## Teaser

Annibaldi et al. show that cIAP-mediated ubiquitylation of RIPK1 kinase suppresses its auto-activation and, in addition, marks it for proteasomal degradation. These results reveal a direct role for ubiquitin in controlling RIPK1 kinase activity and suppressing TNF-mediated cytotoxicity.

## Introduction

Inflammation and cell death are ancient processes of fundamental biological importance that enable survival and adaptation during infection and injury. Tumor necrosis factor (TNF) is a potent inflammatory cytokine that triggers, through its type 1 receptor (TNF-R1), either pro-survival/inflammatory or pro-death signaling pathways in a ubiquitin (Ub)- and phosphorylation-dependent manner (Annibaldi and Meier, 2018). TNF can regulate tissue homeostasis in at least three different ways: (1) activation of nuclear factor κB (NF-κB) and MAPK/JNK-transcriptional programs, (2) induction of caspase-8-dependent apoptosis, or (3) stimulation of receptor-interacting protein kinase (RIPK)-mediated necroptosis (Declercq et al., 2009).

Binding of TNF to TNF-R1 results in the formation of two signaling complexes (Micheau and Tschopp, 2003). Upon TNF ligation, a protein complex assembles on the cytoplasmic tail of TNFR1. This complex, frequently referred to as complex-I, consists of TNF-R1, the adaptors TRADD and TRAF2, the kinase RIPK1, and the E3 ubiquitin (Ub) ligases cellular inhibitor of apoptosis 1 (cIAP1) and cIAP2 (Silke, 2011, Ting and Bertrand, 2016). Within this complex, RIPK1 and other proteins are rapidly conjugated with M1, K11, K48, and K63 Ub linkage types (Dondelinger et al., 2016, Dynek et al., 2010, Gerlach et al., 2011, Peltzer et al., 2016), and cIAP-mediated conjugation of Ub to RIPK1 allows recruitment of the kinase complex TAK1/TAB2/TAB3 and the E3 ligase linear Ub chain assembly complex (LUBAC, composed of HOIL/HOIP/SHARPIN). LUBAC-mediated linear ubiquitylation of different components of complex-I (RIPK1, TRADD, and TNF-R1) subsequently reinforces complex-I and allows efficient recruitment and activation of IKK (composed of NEMO/IKKα/IKKβ), which in turn drives activation of NF-κB (Zinngrebe et al., 2014). While the synthesis of M1- and K63-linked poly-Ub chains play key roles in Ub-dependent assembly of complex-I and the induction of NF-κB target genes that drive inflammation and cell survival following TNF stimulation, the role of K11 and K48 poly-Ub remains largely uncharacterized.

TNF-induced cell death is mediated by an RIPK1-containing secondary complex that is frequently referred to as complex-II or necrosome (Micheau and Tschopp, 2003, Pasparakis and Vandenabeele, 2015, Wang et al., 2008). It is thought that the Ub chains conjugated to RIPK1 by cIAP1/2 and LUBAC in complex-I constitute one of the decisive factors preventing RIPK1 from forming complex-II and limiting its killing potential (Bertrand et al., 2008, Haas et al., 2009, Peltzer et al., 2016). Consistently, genetic deletion of cIAPs completely abrogates RIPK1 ubiquitylation, leading to exaggerated complex-II formation and RIPK1-mediated cell death in response to TNF (Moulin et al., 2012). The interpretation of the role of RIPK1 ubiquitylation in suppressing the cytotoxic potential of RIPK1 is complicated by the fact that loss of cIAPs not only abrogates RIPK1 ubiquitylation, but also interferes with recruitment of LUBAC, TAK1, and IKK. In particular, loss of TAK1 recruitment prevents activation of MK2 and IKK, which in turn regulate the cytotoxic potential of RIPK1 via direct phosphorylation (Dondelinger et al., 2015, Dondelinger et al., 2017, Jaco et al., 2017, Menon et al., 2017). Thus, loss of cIAPs not only interferes with activation of NF-κB, but also abrogates MK2- and IKK-mediated suppression of RIPK1 (Bettermann et al., 2010, O’Donnell et al., 2007, Vandenabeele and Bertrand, 2012).

While it is beyond doubt that cIAPs suppress TNF-induced cell death, how this is achieved remains unclear. The main problem in dissecting cIAP-mediated regulation of TNF-induced cell death has been the fact that the signaling aspect of Ub (recruitment/activation of TAK1, IKK, MK2, and NF-κB-mediated gene induction) and the direct Ub-dependent anti-apoptotic function of cIAPs cannot be separated. We now identified a point mutation in cIAP1 that selectively sensitizes cells to TNF-induced cell death, without interfering with TNF-mediated activation of NF-κB, and IKK- and MK2-mediated phosphorylation of RIPK1. This mutation affects the evolutionarily conserved ubiquitin-associated (UBA) domain of cIAP1. Mice with a knockin mutation in the UBA domain develop normally but are acutely sensitive to TNF-induced systemic inflammatory response syndrome (SIRS), which is caused by enhanced sensitivity to TNF-mediated cell death. Our data are consistent with the notion that the UBA domain is required for Ub-mediated regulation of RIPK1 kinase activity. We find that cIAP1 represses RIPK1 kinase auto-activation via UBA-dependent ubiquitylation of an expanded repertoire of Ub-acceptor lysines of RIPK1. In addition, we find that cIAP1 with a functional UBA domain increases the number of ubiquitylation sites on RIPK1. Moreover, it enhances K48-linked poly-ubiquitylation of RIPK1. Together, this destabilizes active RIPK1 via proteasomal degradation. In the absence of a functional UBA domain, fewer K residues are ubiquitylated and fewer K48-linked chains are present on RIPK1. Together, this causes lethal accumulation of active RIPK1 kinase in response to TNF in *cIAP1*^*UBAmut*^ cells. Our data demonstrate that cIAP-mediated ubiquitylation of RIPK1 directly regulates its kinase activity, independently of the recruitment of IKK and TAK1 kinase complexes.

## Results

### Multivalent Interactions between cIAP1 and TRAF2

**Figure 1:**
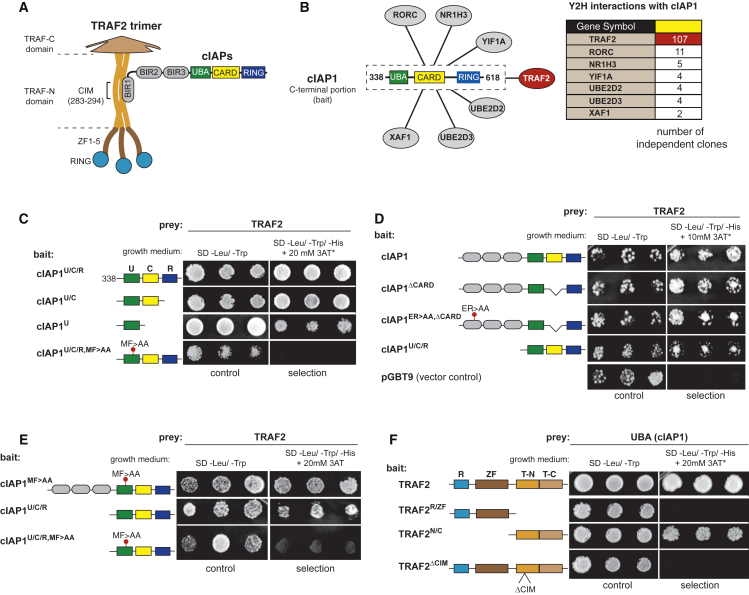
The UBA Domain of cIAP1 Interacts with TRAF2 (A) Schematic representation of the domain architecture of cIAPs and TRAF2, and the interaction between cIAPs and TRAF2. (B) Schematic representation of the putative interaction partners of cIAP1, identified by yeast two-hybrid using the C-terminal portion (encompassing the UBA/CARD/RING region) of cIAP1 as bait. (C–F) Yeast two-hybrid analysis studying the interaction between the indicated cIAP1 fragments and TRAF2 variants. Three single colonies for each cotransformation grown on nonselective (SD-Leu-Trp) or selective medium (SD-Leu-Trp-His, containing the indicated 3AT concentration) are shown.

The BIR1 and RING domains of cIAP1/2 are required for TNF signaling, but little is known about the role of the UBA domain. Because UBA domains often regulate protein activity via protein-protein interactions (Dikic et al., 2009, Hicke et al., 2005, Yagi et al., 2012), we conducted a yeast two-hybrid experiment with the UBA containing C-terminal portion of cIAP1 (cIAP1^U/C/R^) to establish a UBA interactome of cIAP1 (Figures 1A and 1B). This identified known as well as novel cIAP1-binding proteins (Figure 1B). Surprisingly, out of the 137 clones identified, TRAF2 was isolated 107 independent times. While previous work established that TRAF2 binds to the BIR1 of cIAP1 and cIAP2 (Samuel et al., 2006, Vince et al., 2009, Zheng et al., 2010) (Figure 1A), our data suggest that TRAF2 also associates with the C-terminal portion of cIAP1.

To narrow down the region within the UBA-CARD-RING fragment that mediates TRAF2 binding, we determined the ability of truncation mutants to interact with TRAF2. The UBA domain readily interacted with TRAF2 (Figures 1C and S1A), and point mutations in the conserved MGF motif of the hydrophobic patch of the UBA domain (MF > AA) abrogated TRAF2 binding. Consistent with the notion that cIAP1 binds TRAF2 through multivalent interactions via its BIR1 as well as UBA domains, we found that point mutations in either the BIR1 (ER > AA) or UBA (MF > AA) did not abolish the interaction between cIAP1 and TRAF2 in yeast two-hybrid experiments (Figures 1D, 1E, and S1B).

We next mapped the region of TRAF2 that bound to the UBA domain. Surprisingly, the cIAP-interacting motif (CIM) in the TRAF-N domain, which is required for TRAF2 to interact with the BIR1 of cIAP1 (Vince et al., 2009), was also indispensable for UBA binding. Accordingly, deletion of the CIM completely abrogated the interaction between TRAF2 and the UBA domain of cIAP1 (Figures 1F and S1C). These data indicate that cIAP1 contains two surfaces on very distinct spatially separated domains that bind to the same short TRAF2 motif. Because TRAF2 forms trimers (Zheng et al., 2010), we cannot discern whether the UBA and BIR1 bind to the very same CIM of one TRAF2 molecule or to different CIMs of adjacent molecules.

### The UBA Contributes to TRAF2 Binding in Solution and in Cells

**Figure 2:**
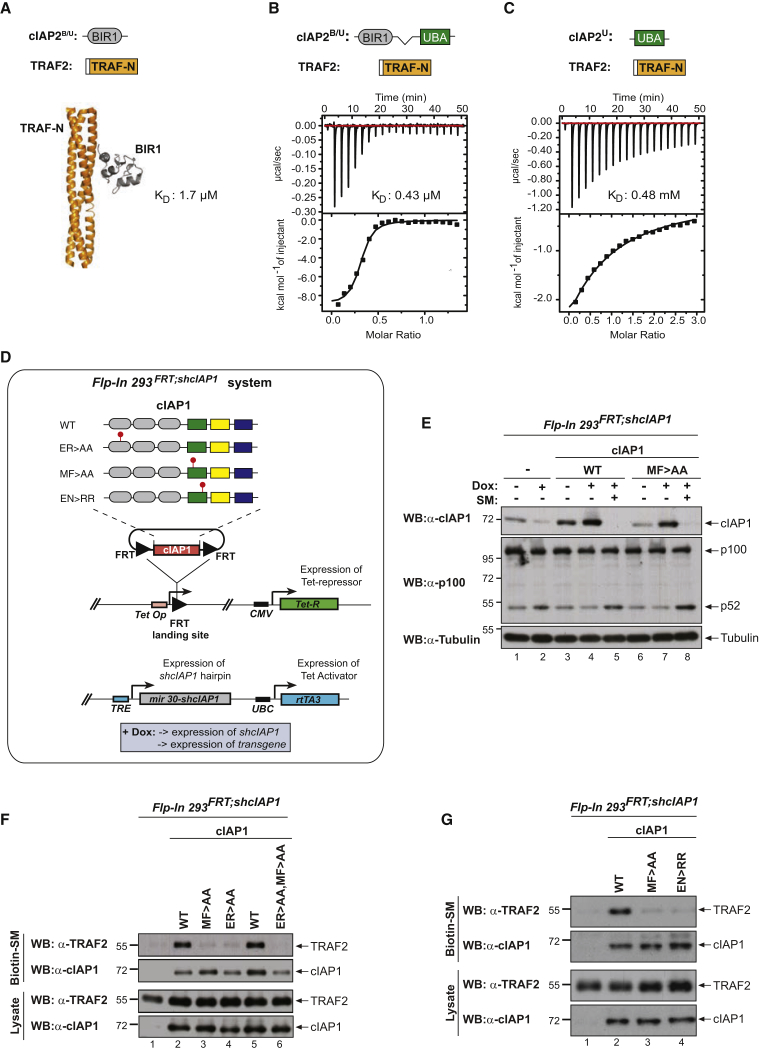
cIAP2 Requires a Functional UBA Domain to Efficiently Interact with TRAF2 (A–C) Binding of the indicated cIAP2 fragments to TRAF2 was measured by isothermal titration calorimetry. K_D_, binding constant. Note the data shown in (A) are from Zheng et al. (2010) and are shown for the purposes of comparison only. (D) Schematic diagram of the *Flp-In*^*TM*^*T-REx*^*TM*^*-HEK293*^*shcIAP1*^ cell system in which endogenous *cIAP1* was knocked down via inducible expression of *mir30*-based shRNA targeting *cIAP1*’s 3′ UTR. These cells also carry a single *FRT* site that allows Flp-mediated integration of transgenes into the same transcriptionally regulatable genomic locus. Expression of the transgene and the *mir30*-based *shcIAP1* are induced following treatment with doxycycline (Dox). *TRE*, tetracycline response element; UBC, ubiquitin promoter; FRT, flippase recognition target; Tet Op, tetracycline operator; Tet-R, tet repressor protein; rtTA3, reverse Tet transactivator (rtTA3). (E) Western blot analysis of *Flp-In* cells treated for 72 hr with Dox (100 ng/mL), to allow expression of the indicated transgenes, followed by treatment with the SMAC mimetic (SM) compound A (100 nM) for 6 hr. (F and G) Biotinylated SM was used to purify IAPs from lysates of *Flp-In* cells that were treated with Dox for 72 hr. TRAF2-binding was then assessed by immunoblotting. In parallel, expression levels of cIAP1 and TRAF2 were controlled by immunoblotting total cell lysates with the respective antibodies. Representative immunoblots are shown of three independent experiments.

To independently corroborate the interaction, we performed isothermal titration calorimetry (ITC) using recombinant cIAP2. cIAP2 was used instead of cIAP1 because structural information of TRAF2/cIAP2 is available, and previous ITC measurements indicate that the BIR1 domains of cIAP1 and cIAP2 bind TRAF2 equivalently (Zheng et al., 2010). Of note, cIAP1’s UBA is 87% similar to that of cIAP2. While the interaction between TRAF2 and the BIR1 domain of cIAP2 exhibits a dissociation constant of 1.7 μM (Zheng et al., 2010), we found that the BIR1 in conjunction with the UBA domain bound to TRAF2 with a significantly higher affinity (0.43 μM) (Figures 2A and 2B). The increase in affinity can be explained by direct binding of the UBA domain to TRAF2, with a dissociation constant of 0.48 mM (Figure 2C).

To determine the importance of the UBA domain for TRAF2 binding, we used *Flp-In*^*TM*^*-Rex*^*TM*^*-*HEK293 (referred to as Flp-In) cells. Prior to transgene insertion, isogenic parental HEK293^*Flp-In;shcIAP1*^ cells bearing a doxycycline-inducible mir30-based short hairpin RNA (shRNA) against the 3′ UTR of endogenous *cIAP1* were generated and reconstituted with either wild-type (WT) cIAP1 or the indicated mutants (Figure 2D). Because HEK293^*Flp-In*^ cells do not express detectable levels of cIAP2 (data not shown), this system ensures single-copy insertion and equal expression levels of untagged cIAP1 proteins without interference from endogenous cIAPs. Expression of the doxycycline-inducible *cIAP1* shRNA in parental cells reduced cIAP1 to an almost undetectable level and resulted in concomitant activation of the non-canonical NF-κB pathway (Figure 2E). Cells reconstituted with either a WT or a UBA mutant version of cIAP1 exhibited comparable levels of cIAP1, indicating that the UBA mutation did not affect protein stability. Moreover, cIAP1^MF > AA^ suppressed activation of the non-canonical NF-κB pathway (Figure 2E) and underwent SMAC mimetic (SM)-induced auto-ubiquitylation and degradation, indicating that cIAP1^MF > AA^ is able to ubiquitylate NIK and itself. Biotinylated SM readily co-purified TRAF2 with WT cIAP1 (Figure 2F). In contrast, and consistent with earlier reports (Samuel et al., 2006, Vince et al., 2009, Zheng et al., 2010), we found that mutation of the BIR1 (cIAP1^ER > AA^) almost completely abolished the binding of cIAP1 to TRAF2 (Figure 2F). Interestingly, mutation of the UBA domain, via either alteration of the MGF motif (MF > AA) or substitutions of E401 and N428 to RR (EN > RR), which disrupt UBA-mediated protein:protein interactions (Budhidarmo and Day, 2014), likewise impaired TRAF2 binding (Figures 2F and 2G). While cIAP1-BIR1^ER > AA^ and -UBA^MF > AA^ mutants retained some binding to TRAF2, combined mutation (ER > AA/MF > AA) completely abrogated the interaction between cIAP1 and TRAF2 (Figure 2F). Together, these data corroborate the notion that TRAF2 interacts with cIAP1 via its BIR1 and UBA domain.

### The UBA Domain Is Dispensable for Development and Regulation of NF-κB

**Figure 3:**
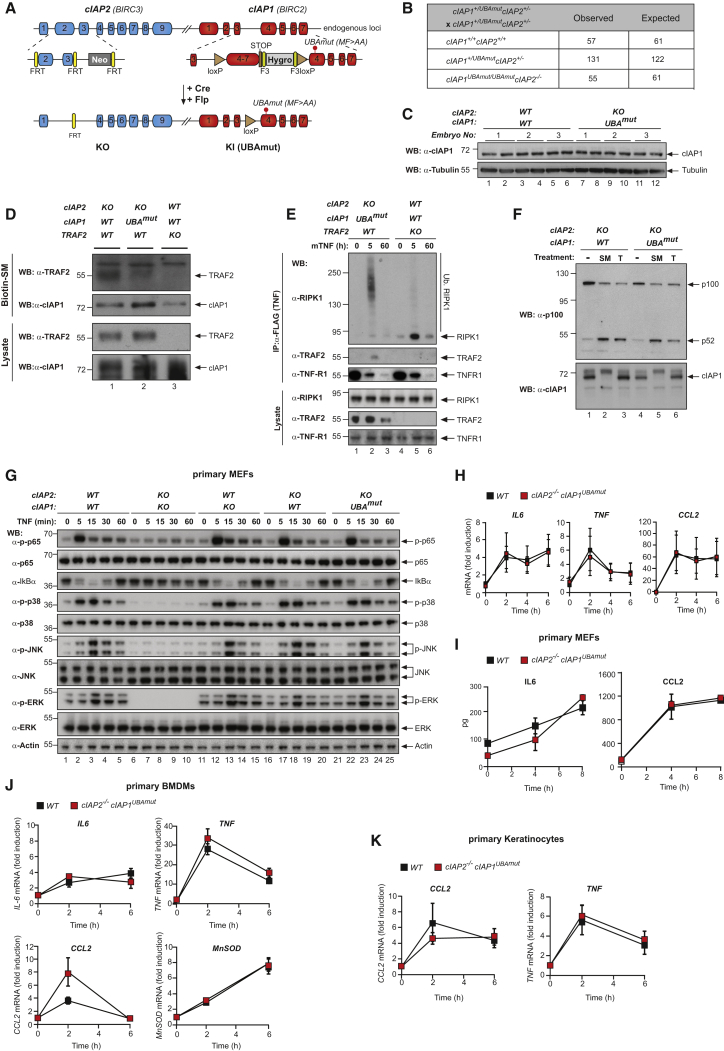
Mice with a Knockin Mutation in the UBA Domain Develop Normally and Do Not Exhibit Defects in the Canonical and Non-canonical NF-κB Activation (A) Gene targeting strategy for the generation of mice with conditional deletion of *cIAP2* and conditional mutation of the UBA domain of *cIAP1*. Exon 2 and 3 of *cIAP2* were flanked by FRT sites. To generate the UBA mutation, M396 and F398 were mutated to A396 and A398, respectively. A targeting vector containing a lox-P flanked-minigene spanning exon 4 to 7 of *cIAP1* followed by a stop sequence and a hygromycin resistance sequence was used to ensure the WT expression of cIAP1 and therefore the conditional expression of the UBA mutation. (B) Expected and observed numbers of mice from crosses with the respective genotypes. (C) Western blot analysis of cIAP1 protein levels of WT and *cIAP1*^*UBAmut*^ MEFs obtained from three different embryos. (D) Biotinylated SM was used to purify IAPs from lysates of *cIAP2*^−/−^ and *cIAP1*^*UBAmut*^ MEFs and TRAF2 binding was then assessed by immunoblotting. In parallel, expression levels of cIAP1 and TRAF2 were controlled by immunoblotting total cell lysates with the indicated antibodies. (E) Purification of the TNF-receptor signaling complex (complex-I) from immortalized MEFs. Cells of the indicated genotypes were treated with FLAG-TNF for 0, 5, and 60 min. Cell lysates were then subjected to FLAG IP followed by western blot analysis with the indicated antibodies. Representative images of at least three independent experiments are shown. (F) Western blot analysis of *cIAP2*^−/−^ and *cIAP1*^*UBAmut*^ MEFs treated with SM (100 nM) or TWEAK for 6 hr, followed by western blot analysis using the indicated antibodies. (G) Western blot analysis of MEFs with the indicated genotypes treated with TNF and harvested at the indicated times points. (H and I) The presence of relative mRNA levels (H) and cytokines in the culture media (I) of MEFs treated with TNF (10 ng/mL) for the indicated time points were analyzed by RT-PCR and ELISA, respectively. Graphs show mean ± SD; n = 3 independent biological repeats. (J and K) Primary WT and *cIAP1*^*UBAmut*^ BMDMs (J) and keratinocytes (K) were treated with TNF (10 ng/mL) for 2 and 6 hr, and mRNA levels of the indicated cytokines were measured by RT-PCR. Graphs show mean ± SD, n = 3 independent biological repeats.

To study the function of the UBA domain of cIAP1 *in vivo*, we generated a conditional knockin mouse bearing the MF > AA mutation in the absence of cIAP2 (Figure 3A). Previous work indicated that cIAP1 and cIAP2 function redundantly to each other (Conte et al., 2006, Conze et al., 2005, Moulin et al., 2012). Therefore, we generated the conditional *cIAP1*^*UBAmut*^ mouse from an embryonic stem cell (ESC) clone that previously had been targeted at the *cIAP2* locus (Moulin et al., 2012). These doubly targeted animals (*cIAP2*^−/−^*cIAP1*^*UBAmut*^) are subsequently referred to as *cIAP1*^*UBAmut*^. *cIAP1*^*UBAmut*^ mice were weaned at the expected Mendelian ratio (Figure 3B) and were indistinguishable from their WT counterparts (Figures S2A and S2B). Additionally, these mice had an overtly normal immune system (Figure S2C). Primary mouse embryonic fibroblasts (MEFs) from WT and *cIAP1*^*UBAmut*^ embryonic day 13.5 (E13.5) embryos exhibited the same cIAP1 protein levels (Figure 3C), indicating that the UBA mutation had no impact on the stability of cIAP1. As expected, these cells exhibited undetectable levels of *cIAP2* mRNA (Figure S2D). To verify whether the UBA mutation affected cIAP1’s E3 ligase function, we tested the ability of SM to stimulate auto-ubiquitylation and degradation of cIAP1^UBAmut^. We found that the behavior of cIAP1^UBAmut^ was indistinguishable from its WT counterpart (Figure S2E).

To test the binding of cIAP1^UBAmut^ to TRAF2, we purified cIAP1 using biotinylated SM. Significantly less TRAF2 was co-purified with cIAP1 in *cIAP1*^*UBAmut*^ cells, while no TRAF2 binding was observed in *TRAF2*^−/−^ cells (Figure 3D). Importantly, however, while this immunoprecipitation (IP) setting reveals a weakened association between cIAP1^UBAmut^ and TRAF2, cIAP1^UBAmut^ and TRAF2 are perfectly capable of interacting with each other in a physiological meaningful way because the UBA mutation of cIAP1 does not phenocopy loss of TRAF2 (Figure 3E). Accordingly, RIPK1 is readily poly-ubiquitylated in complex-I from *cIAP1*^*UBAmut*^ cells, while RIPK1 ubiquitylation was lost in *Traf2* knockout (KO) cells (Figure 3E). Moreover, cIAP1:TRAF2-mediated regulation of NIK, and suppression of non-canonical NF-κB, was normal in *cIAP1*^*UBAmut*^ cells (Figure 3F). In contrast, depletion of cIAP1^WT^ and cIAP1^UBAmut^ by SM or depletion of TRAF2 by TWEAK activated (Vince et al., 2008) non-canonical NF-κB (Figure 3F). As TRAF2 is essential to bring cIAPs to NIK, these data demonstrate that cIAP1^UBAmut^:TRAF2 association is sufficiently strong *in vivo* to target NIK for degradation. Additionally, a functional UBA domain of cIAP1 was dispensable for timely TNF-induced phosphorylation of p65, degradation of IκB, phosphorylation of MAPKs (Figures 3G and S2F), and the production of cytokines in primary MEFs, bone marrow-derived macrophages (BMDMs), and keratinocytes (Figures 3H–3K). Taken together, our data demonstrate that cIAP1^UBAmut^ retains E3 ligase activity, and a functional UBA domain is dispensable for embryonic development or routine tissue homeostasis. Additionally, we conclude that a functional UBA domain of cIAP1 is not required for Ub-dependent formation of complex-I, activation of the canonical NF-κB pathway, and suppression of non-canonical NF-κB signaling.

### *cIAP1*^*UBAmut*^ Mice Develop Normally but Are Acutely Sensitive to TNF-Induced Systemic Inflammatory Response Syndrome

**Figure 4:**
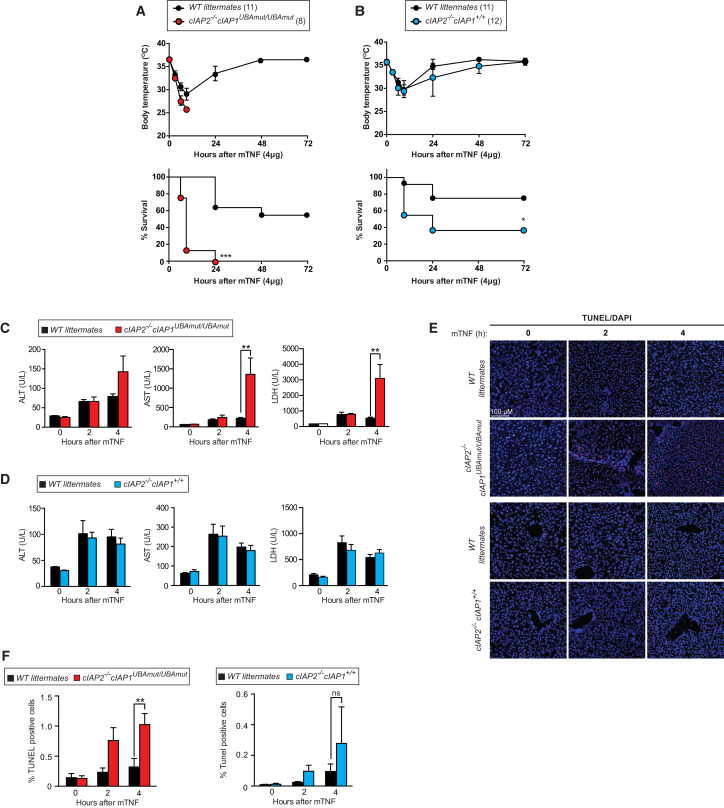
Mice with a Knockin Mutation in the UBA Are Acutely Sensitive to TNF-Induced Systemic Inflammatory Response Syndrome (A and B) Body temperature and survival of WT (A, n = 11; B, n = 12) and corresponding littermate *cIAP1*^*UBAmu*t^ (A, n = 8) or *cIAP2*^−/−^ (B, n = 11) mice injected with 4 μg/20 g body weight of mTNF. Data are representative of two independent experiments. Error bars represent SD. Survival curves were compared using log-rank Mantel-Cox test (^∗^p < 0.05, ^∗∗∗^p < 0.001). (C and D) Plasma samples of WT and *cIAP1*^*UBAmut*^ (C) or *cIAP2*^−/−^ (D) mice were collected at the indicated time points following challenge with mTNF (4 μg/20 g body weight, intravenously [i.v.]) and analyzed for activities of LDH, AST, and ALT. n = 4 per time point and genotype. Data are presented as mean ± SD, ^∗∗^p < 0.01; statistics were performed using two-way ANOVA. (E and F) TUNEL staining (E) and quantification (F) of liver sections of WT and *cIAP1*^*UBAmut*^ mice used in (C) and (D). Data in (A) and (B) were obtained from two sets of animals, while the data shown in (C)–(F) were obtained from a third set of animals. Graphs show mean ± SD, ^∗∗^p < 0.01; statistics were performed using two-way ANOVA.

Next, we tested the response of *cIAP1*^*UBAmut*^ mice to TNF challenge. Injection of TNF provokes systemic inflammation that is driven by RIPK1 kinase-dependent cell death (Duprez et al., 2011) and resembles clinical SIRS (Tracey et al., 1986). Strikingly, *cIAP1*^*UBAmut*^ mice were much more sensitive to TNF-induced SIRS than WT and *cIAP2*^−/−^ counterparts. Accordingly, following administration of a dose of murine TNF as low as 4 μg/20 g of body weight, *cIAP1*^*UBAmut*^ mice exhibited a dramatic drop in body temperature and significant increase in morbidity (Figures 4A and 4B). TNF-treated *cIAP1*^*UBAmut*^ mice also had significantly elevated levels of aspartate transaminase (AST), alanine transaminase (ALT), and lactate dehydrogenase (LDH) in the plasma, indicating liver and tissue damage (Figures 4C and 4D). Consistently, livers from *cIAP1*^*UBAmut*^ mice had higher numbers of TUNEL-positive cells than *cIAP2*^−/−^ or WT littermate control mice (Figures 4E and 4F). Collectively, these data demonstrate that a functional UBA domain in cIAP1 is required to protect mice from the lethal effects of TNF.

### Mutation in the UBA Domain Switches the TNF Response to Cell Death

**Figure 5:**
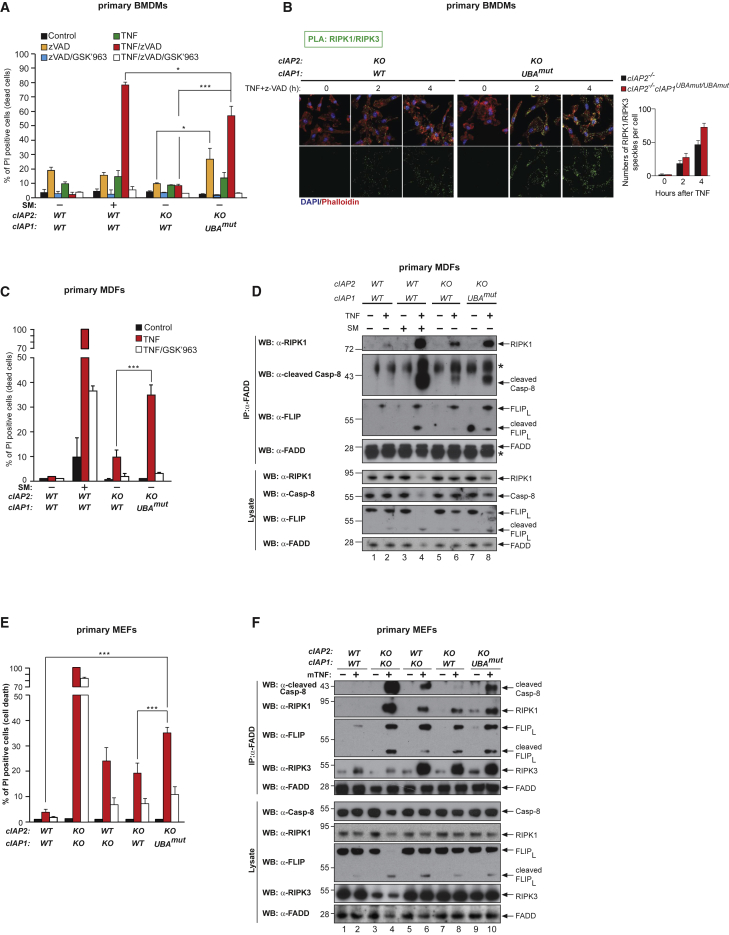
Mutation in the UBA Domain Switches the TNF Response to Cell Death (A, C, and E) Primary BMDMs (A), MDFs (C), and MEFs (E) of the indicated genotypes were treated as shown (TNF 100 ng/mL, GSK′963 100 nM, SM 100 nM, for BMDMs TNF 1 ng/mL) for 24 hr followed by quantification of propidium iodide (PI)-positive cells. Data are presented as mean ± SD, n > 3, ^∗^p < 0.05, ^∗∗∗^p < 0.001; statistics were performed using two-way ANOVA. (B) PLA of primary BMDMs from *cIAP2*^−/−^ and *cIAP1*^*UBAmut*^ animals using RIPK1 and RIPK3 antibodies. Cells were stimulated with 1 ng/mL TNF for the indicated time points. The graph to the side indicates the quantification of RIPK1/RIPK3 PLA speckles. The graph shows mean ± SD. (D and F) Primary MDFs (D) and MEFs (F) of the indicated genotypes were treated for 4 hr as indicated (TNF 100 ng/mL, z-VAD-FMK 10 μM), followed by FADD IP and western blot analysis for the indicated proteins. Images are representative of three independent experiments. Graphs show mean ± SD, ^∗^p < 0.05, ^∗∗∗^p < 0.001.

Next, we examined the role of the UBA domain in repressing TNF-induced cell death in primary BMDMs, mouse dermal fibroblasts (MDFs), and MEFs. TNF treatment did not induce substantial cell death in either WT or cIAP2-deficient cells; however, it was a potent cell death stimulus in *cIAP1*^*UBAmut*^ cells (Figures 5A, 5C, 5D, S3A, and S3B). This cell death was RIPK1 kinase dependent because treatment with the selective RIPK1 inhibitor GSK′963 (Berger et al., 2015) suppressed TNF killing. Likewise, primary *cIAP1*^*UBAmut*^ BMDMs were exquisitely more sensitive to RIPK1-mediated TNF-induced necroptosis than BMDMs from either WT littermates or single targeted *cIAP2*^−/−^ animals (Figure 4A). Consistently, increased association of RIPK1 with RIPK3 was detected by proximity ligation assay (PLA) (Figure 5B). Primary MDFs and MEFs instead seemed to die by apoptosis because RNAi-mediated depletion of MLKL had no apparent effect on TNF-induced cell death in *cIAP1*^*UBAmut*^ MEFs (Figure S3D). Indeed, TNF stimulation of *cIAP1*^*UBAmut*^ MDFs and MEFs caused elevated levels of caspase activity (Figure S3E), which was accompanied with enhanced complex-II formation (Figures 5D and 5F), and cleavage and activation of caspase-8 and caspase-3 (Figure S3F). Importantly, *cIAP1*^*UBAmut*^ MEFs, prepared from multiple embryos within the same litter and between litters, behaved in the same manner (Figures S3A–S3C). Together, these data suggest that the UBA domain allows cIAP1 to inhibit TNF-induced death.

### The UBA Directly Regulates RIPK1 Ubiquitylation

**Figure 6:**
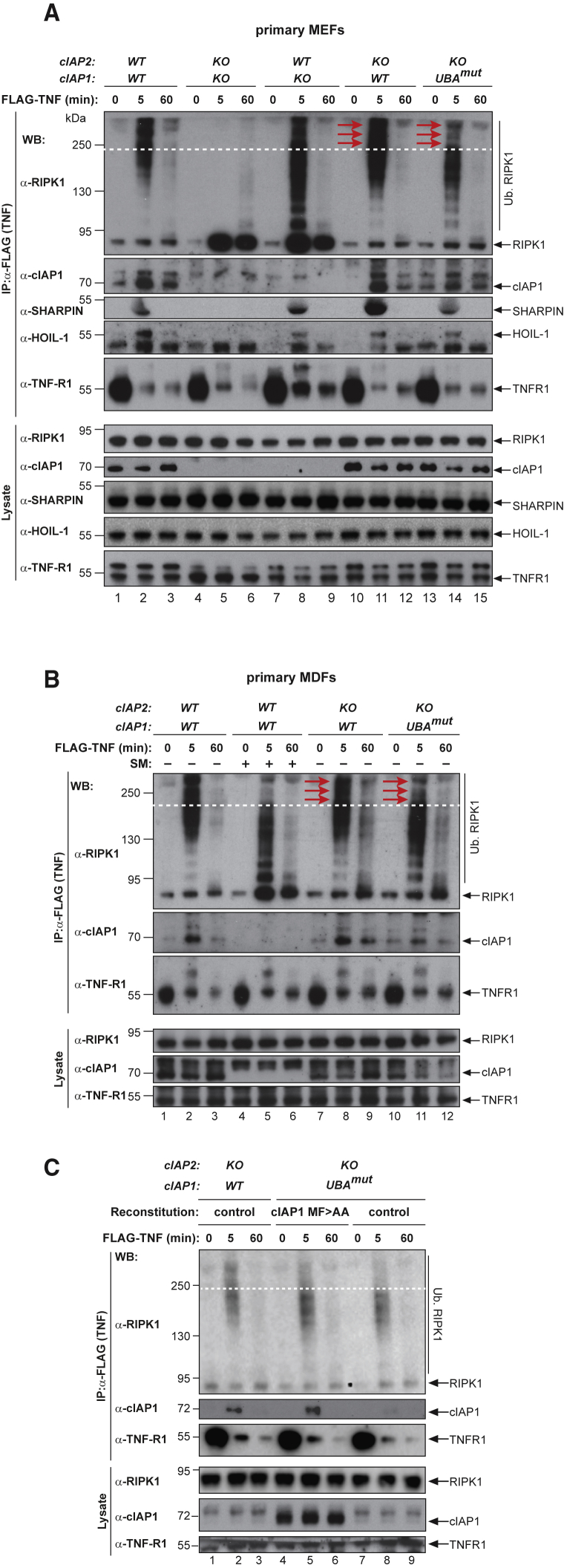
The UBA Directly Regulates RIPK1 Ubiquitylation (A and B) Purification of the TNF-R1 signaling complex (complex-I) from primary MEFs (A) and MDFs (B). Cells of the indicated genotypes were treated with FLAG-TNF for 0, 5, and 60 min. Cell lysates were then subjected to FLAG IP followed by western blot analysis with the indicated antibodies. Representative images of three independent experiments are shown. (C) Purification of the TNF-R1 signaling complex (complex-I) from immortalized *cIAP2*^−/−^ and *cIAP1*^*UBAmut*^ MEFs reconstituted either with empty vector (control) or a doxycycline-inducible construct encoding *cIAP1*^*UBAmut*^.

Because ubiquitylation of RIPK1 and other components of complex-I represses RIPK1-dependent formation of complex-II, we analyzed complex-I formation in MDFs and MEFs. As previously reported, deficiency of cIAP1 and cIAP2 prevented ubiquitylation of RIPK1 and the recruitment of the LUBAC components SHARPIN and HOIL-1 5 min after TNF stimulation (Figure 6A) (Haas et al., 2009). We found no evidence for defective ubiquitylation of RIPK1 in complex-I in single-targeted *cIAP2*^−/−^, but *cIAP1*-deficient MEFs had significantly lower molecular weight-modified forms of RIPK1 and substantial levels of non-modified RIPK1. *cIAP1*^*UBAmut*^ cells, however, displayed a marked and reproducible decrease in the extent of high molecular weight RIPK1 ubiquitylation (red arrows) in complex-I in both MEFs and MDFs (Figures 6A, 6B, and S4A), compared to *cIAP2*^−/−^ and WT cells. Importantly, the extent of non-modified RIPK1 in complex-I was indistinguishable between WT, *cIAP2*^−/−^, and *cIAP1*^*UBAmut*^ cells (Figures 6A and 6B), suggesting that equivalent amounts of RIPK1 undergo Ub modifications in these three genotypes. Because the same amount of RIPK1 is being ubiquitylated in WT, *cIAP2*^−/−^, and *cIAP1*^*UBAmut*^ cells, but overall ubiquitylation of RIPK1 seems to be affected in *cIAP1*^*UBAmut*^ cells compared to WT and *cIAP2*^−/−^ cells, we conclude that RIPK1 undergoes Ub modifications that are distinct from those observed in WT, *cIAP1*^−/−^, or *cIAP2*^−/−^ cells. Although the UBA mutation resulted in reduced levels of ubiquitylated RIPK1 in complex-I, this had no apparent effect on the kinetics of the recruitment of other components of the TNF-RSC such as SHARPIN and HOIL-1 (Figures 5A and 5B).

Next, we addressed whether the weakened association of cIAP1^UBAmut^ with TRAF2 might lead to decreased recruitment of cIAP1 to complex-I and decreased ubiquitylation mediated by cIAP1 (Figures 6A and 6B). To this end, we increased the amount of cIAP1^UBAmut^ to the levels of WT cIAP1 in complex-I using a doxycycline-inducible reconstitution approach. This allowed us to induce the expression of cIAP1^UBAmut^ so that a comparable amount of cIAP1^UBAmut^ protein was present in complex-I as in WT or *cIAP2* KO cells (Figure 6C). Even though the levels of cIAP1^UBAmut^ in complex-I were comparable to cIAP1 WT, this did not “normalize/correct” the ubiquitylation pattern of RIPK1, nor did it have an impact on the recruitment of unmodified RIPK1 in complex-I (Figure 6C). This demonstrates that the different smearing pattern of ubiquitylated RIPK1 in *cIAP1*^*UBAmut*^ cells is not due to impaired recruitment of cIAP1 to complex-I, and, therefore, is not TRAF2 binding dependent but merely UBA dependent.

### A Functional UBA Is Required for Efficient K48 Ubiquitylation and Degradation of RIPK1 in Complex-I

**Figure 7:**
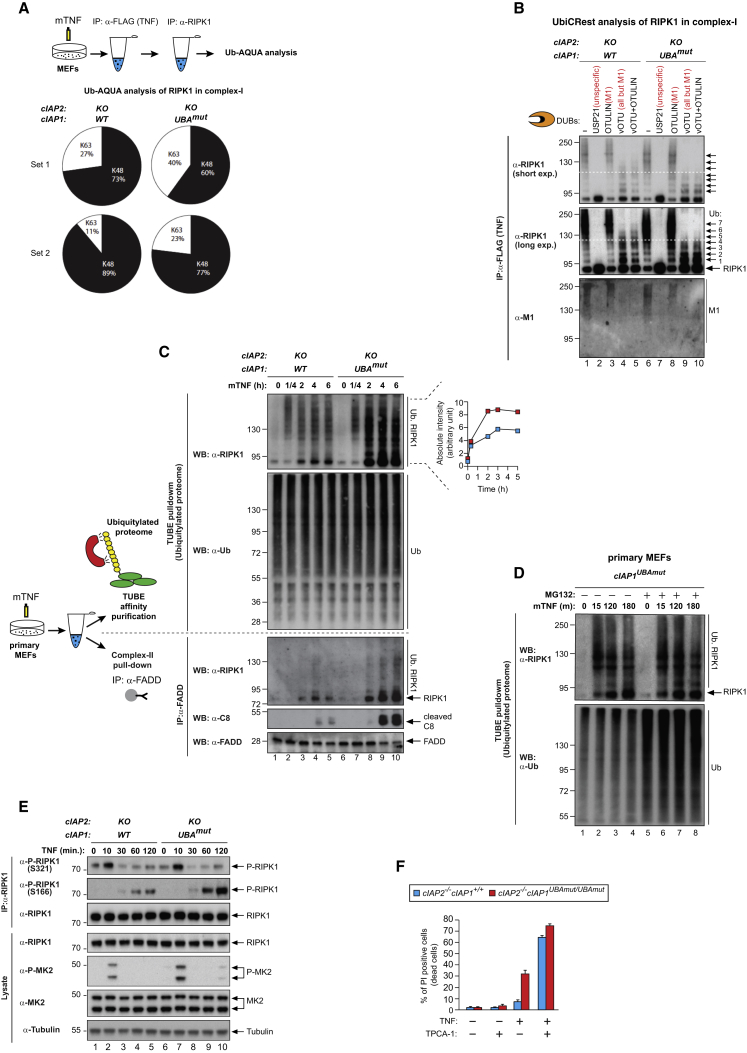
UBA-Dependent Ubiquitylation of RIPK1 Represses Its Kinase Activity and Facilitates RIPK1 Degradation in Response to TNF (A) Absolute quantification (AQUA)-based mass spectrometry of Ub chain linkage types on RIPK1 in complex-I. The scheme indicates the double purification strategy to isolate RIPK1 in complex-I. Pie charts indicate the Ub linkage types detected on RIPK1 in complex-I from *cIAP2*^−/−^ and *cIAP1*^*UBAmut*^ MEFs in two independent replicates, 5 min following TNF stimulation. Values were normalized to the total amount of poly-Ub chains. (B) UbiCRest analysis of ubiquitylated RIPK1 in complex-I. Complex-I was purified from *cIAP2*^−/−^ and *cIAP1*^*UBAmut*^ MEFs using FLAG-TNF as affinity reagent. Immuno-complexes were then subjected to UbiCRest analysis using the indicated DUBs followed by western blot analysis for RIPK1. (C) *cIAP2*^−/−^ and *cIAP1*^*UBAmut*^ MEFs were treated with TNF (100 ng/mL) for the indicated time points. Lysates were then split in two and subjected either to FADD IP (complex-II) or TUBE pull-down (ubiquitylated proteome), followed by western blot analysis with the indicated antibodies. Representative images of three independent experiments are shown. (D) *cIAP1*^*UBAmut*^ MEFs were incubated for 1 hr with MG132 (20 μM) or left untreated and then subjected to TNF stimulation for the indicated time points. TUBE pull-down was then carried out on cell lysates followed by western blot analysis with the indicated antibodies. (E) IP of RIPK1 in *cIAP2*^−/−^ and *cIAP1*^*UBAmut*^ MEFs followed by immunoblotting analysis with the indicated antibodies. (F) *cIAP2*^−/−^ and *cIAP1*^*UBAmut*^ MDFs were treated as indicated for 24 hr (TNF 100 ng/mL, TPCA-1 1 μM) followed by quantification of PI-positive cells. Graphs show mean ± SD.

To provide a robust analysis of the composition of Ub linkage types on RIPK1 in *cIAP2*^−/−^ and *cIAP1*^*UBAmut*^ cells, we employed absolute quantification (AQUA)-based mass spectrometry analysis of Ub chain composition (Ordureau et al., 2015) on complex-I-derived RIPK1. To this end, we performed two consecutive IPs, first of complex-I (FLAG-TNF) and then of RIPK1 (Figure 7A), followed by AQUA-based absolute quantification of chain types using mass spectrometry. Our analysis revealed that K48-linked chains on RIPK1 were reproducibly less abundant (>10% reduction) in *cIAP1*^*UBAmut,cIAP2*−/−^ mutant cells compared to *cIAP2*^−/−^ (Figures 7A and S5A). Besides K63-linked Ub chains, no other linkage types were reproducibly detected on RIPK1 in MEFs, although other groups previously reported the presence of K11- and M1-linked chains on RIPK1, using different cell types and conditions (Dynek et al., 2010, Gerlach et al., 2011). Calculations from the AQUA-based mass spectrometry experiment indicated that the majority of Ub is conjugated in the form of mono-Ub moieties rather than chains, and the actual chains on RIPK1 are surprisingly short, even in the control situation (Figure S5A). Given that the overall smearing pattern is reduced in *cIAP1*^*UBAmut*^ cells, this suggests not only that K48 ubiquitylation is affected, but also that the occupancy of Ub-acceptor K residues is altered.

To study the occupancy of Ub-acceptor lysines of RIPK1, we analyzed ubiquitylated RIPK1 from complex-I using Ub chain restriction (UbiCRest) (Hospenthal et al., 2015) (Figure 7B). To this end, we used a combination of deubiquitylating enzymes (vOTU/OTULIN) that remove all chain types but leave the most proximal Ub attached to RIPK1. vOTU hydrolyzes all Ub-linkage types except M1-linked chains, which can be cleaved by OTULIN. Incubation with vOTU/OTULIN revealed a reduction in the Ub-site occupancy in *cIAP1*^*UBAmut*^ compared to *cIAP2*^−/−^ (Figure 7B). The reduced Ub occupancy of RIPK1 might help to explain the shift toward the lower molecular weights of ubiquitylated RIPK1 in *cIAP1*^*UBAmut*^ cells.

Because K48-linked chains as well as poly-mono-ubiquitylation can target proteins for degradation (Lu et al., 2015), we tested whether the combined reduction in poly- and mono-ubiquitylation of RIPK1 affects the stability of RIPK1 in complex-I. Using tandem Ub-binding entities (TUBEs) (Hjerpe et al., 2009), which allow isolation of poly-ubiquitylated proteins, we found that the levels of ubiquitylated RIPK1 dramatically accumulated in *cIAP1*^*UBAmut*^ cells compared to WT cells over a 6-hr time period following TNF treatment (Figure 7C). Importantly, TNF-induced accumulation of ubiquitylated RIPK1 in *cIAP1*^*UBAmut*^ cells coincided with a significant increase in formation of complex-II and activation of caspase-8 (Figure 7C). This demonstrates that the UBA domain of cIAP1 represses lethal accumulation of RIPK1, most likely by facilitating efficient poly-mono as well as K48-mediated ubiquitylation and degradation of RIPK1, which would lower the number of “seeding” molecules for formation of complex-II (Jaco et al., 2017). Importantly, while ubiquitylated RIPK1 accumulated over time in TNF-treated *cIAP1*^*UBAmut*^ cells, treatment of *cIAP1*^*UBAmut*^ cells with proteasome inhibitors (MG132) did not result in a further increase in RIPK1 accumulation (Figure 7D), corroborating the notion that the stabilization effect is due to the UBA mutation. In contrast, proteasome inhibition in WT cells resulted in a substantial accumulation of RIPK1 in the ubiquitylated proteome (Figure S5A), indicating that under normal conditions RIPK1’s stability is regulated, at least in part, in a Ub- and proteasome-dependent fashion. Of note, because RIPK1 forms amyloid-like structures upon activation, the TUBE-based experiment also purifies non-ubiquitylated RIPK1 that is bound to ubiquitylated RIPK1. Moreover, using a broad-spectrum DUB inhibitor (PR619), we found no evidence for a role of the UBA domain in shielding ubiquitylated RIPK1 from DUB digestion (Figures S5B and S5C).

### UBA-Dependent Ubiquitylation of RIPK1 Represses Its Kinase Activity

TNF-induced activation of IKK and MK2 directly suppresses the kinase activity and cytotoxic potential of RIPK1 (Dondelinger et al., 2015, Dondelinger et al., 2017, Jaco et al., 2017, Menon et al., 2017). In particular, MK2 directly phosphorylates mouse RIPK1 at serine (S)321 and S336 (S320 and S335 in human), which in turn suppress RIPK1 auto-activation at S166 (Dondelinger et al., 2017, Jaco et al., 2017, Menon et al., 2017). Because *cIAP1*^*UBAmut*^ cells are sensitized to RIPK1 kinase-mediated cell death in response to TNF, we addressed whether the UBA mutation affects MK2-mediated suppression of RIPK1 kinase activity. RIPK1 IP from TNF-treated *cIAP2*^−/−^ and *cIAP1*^*UBAmut*^ cells revealed a strong increase in auto-phosphorylation at S166 in *cIAP1*^*UBAmut*^ cells, which is entirely consistent with the notion that TNF causes auto-activation of RIPK1 and RIPK1 kinase-dependent cell death in these cells (Figure 7E). However, activation of MK2 and MK2-mediated phosphorylation of S321 appeared normal in *cIAP1*^*UBAmut*^ cells, demonstrating that activation of RIPK1 kinase activity in *cIAP1*^*UBAmut*^ cells was MK2 independent. Activation of IKK and NF-κB-mediated expression of target genes was equivalent in WT, *cIAP2*^−/−^, and *cIAP1*^*UBAmut*^ cells (Figures 3G–3J), suggesting that IKK-mediated regulation of RIPK1 was not perturbed in *cIAP1*^*UBAmut*^ cells. Consistent with this view, we find that inhibition of IKK with TPCA-1 further sensitized *cIAP1*^*UBAmut*^ cells to TNF killing (Figure 7F). Together, our data are consistent with a model whereby cIAP1 regulates RIPK1 kinase activity not only by activating downstream kinases, such as IKK and MK2, but also by directly repressing RIPK1 kinase activity via Ub-dependent inactivation.

## Discussion

Ub-mediated inactivation of RIPK1 has long been postulated to contribute to the regulation of cytokine-induced cell death (Ea et al., 2006, Moquin et al., 2013, O’Donnell et al., 2007). However, detailed insights into the determinants and the actual molecular and functional consequences of RIPK1 ubiquitylation have not been demonstrated. Here, we show that the UBA domain of cIAP1 interacts with TRAF2 and is required for proper regulation of RIPK1 kinase activity. In the absence of a functional UBA domain, more active RIPK1 kinase accumulates in response to TNF, causing RIPK1 kinase-mediated cell death and systemic inflammatory response syndrome.

UBA-dependent ubiquitylation of RIPK1 seems to regulate RIPK1 kinase activity through two potentially interconnected mechanisms: first, the UBA contributes to optimal Ub occupancy of RIPK1. This is evident as fewer lysine residues are conjugated to Ub in cells from *cIAP1*^*UBAmut*^ animals. While this reduction in ubiquitylation of RIPK1 has no effect on its scaffolding function, such as activation of TAK1, IKK, MK2, and NF-κB, and the production of cytokines, reduced RIPK1 ubiquitylation compromises regulation of RIPK1 kinase activity, leading to enhanced RIPK1 auto-phosphorylation and formation of complex-II. Second, the UBA domain of cIAP1 also influences the cytotoxic potential of RIPK1 by targeting it for proteasomal degradation.

Although the overall difference in K48 ubiquitylation of RIPK1 5 min after TNF treatment was reproducibly small, this causes a significant alteration of RIPK1’s stability as active, ubiquitylated RIPK1 accumulates over time in *cIAP1*^*UBAmut*^ cells. Multiple Ub modifications, which include short Ub chains, constitute a powerful degradation signal (Lu et al., 2015). Hence, it is likely that the combined reduction in poly- and mono-ubiquitylation of RIPK1 ultimately contributes to the increased protein stability of RIPK1 in complex-I from *cIAP1*^*UBAmut*^ cells, leading to exacerbated RIPK1 kinase activity in response to TNF. Thus, under normal conditions, the conjugation of Ub to RIPK1 might impede its auto-activation and, in addition, mark it for proteasomal degradation, thereby limiting accumulation of cytotoxic RIPK1. Nevertheless, cIAP1 clearly also suppresses the cytotoxic potential of RIPK1 in a UBA-independent manner. Thus, cIAP1 also represses RIPK1 auto-activation by facilitating Ub-mediated recruitment of LUBAC, and activation of IKK and MK2. This enables IKK- and MK2-mediated regulation of RIPK1 via inhibitory phosphorylation (Dondelinger et al., 2015, Dondelinger et al., 2017, Jaco et al., 2017, Menon et al., 2017). Our current data are consistent with a revised model for TNF signaling whereby RIPK1’s kinase activity is suppressed through both Ub-mediated phosphorylation of RIPK1 by IKK and MK2 as well as by direct Ub-mediated inactivation and degradation of RIPK1. However, we cannot formally exclude the possibility that the UBA also enables the recruitment of a yet to be identified kinase, which might repress RIPK1.

Unexpectedly, we find that the UBA domain of cIAP1 contributes to efficient TRAF2 binding. Consistent with the notion that cIAP1 interacts with TRAF2 through a multivalent interaction via its BIR1 as well as UBA domain, we find that point mutations in either the BIR1 or UBA domain weaken the interaction between cIAP1 and TRAF2. Although cIAP1^UBAmutant^ proteins bind less well to TRAF2 in coIP studies under resting conditions, under *in vivo* settings this association is sufficient to maintain TNF-induced activation of NF-κB or support TRAF2/TRAF3-mediated degradation of NIK. Importantly, despite the fact that UBA mutant cIAP1 interacts less efficiently with TRAF2 under IP conditions, the reduced ubiquitylation of RIPK1 in complex-I observed in *cIAP1*^*UBAmutant*^ cells is not due to impaired cIAP1 recruitment. This is evident as elevating the recruitment of cIAP1^UBAmut^ into complex-I does not “normalize/correct” the ubiquitylation pattern of RIPK1. Further evidence is provided by the fact that the UBA mutation of cIAP1 does not phenocopy loss of TRAF2. Accordingly, RIPK1 is readily ubiquitylated in complex-I in cells from *cIAP1*^*UBAmut*^ animals. In contrast, RIPK1 ubiquitylation in complex-I is completely lost in *Traf2*^−/−^ cells. Taken together, our data demonstrate that cIAP1^UBAmut^ retains E3 ligase activity, and the UBA domain is dispensable for embryonic development, routine tissue homeostasis, or NF-κB regulation. Thus, the elevated sensitivity to the cytotoxic potential of TNF cannot be explained by the regulation of NF-κB, IKK, or MK2. We propose that the UBA mutation provides rare insight into the protein architecture of complex-I. We suggest that the UBA contributes to the proper positioning of the RING domain of cIAP1 within complex-I so that the Ub-loaded E2 enzyme can optimally transfer ubiquitin to RIPK1.

As TNF is a key player in the cytokine network that supports inflammation-associated cancer and cancer-related inflammation (Mantovani et al., 2008), it will be important to gain a better understanding of the checkpoints that control life and death decisions in response to TNF. A better understanding of such checkpoints could lead to new approaches for the treatment of chronic inflammatory diseases that are fueled by aberrant RIPK1-induced cell death, and/or reveal novel strategies for anti-cancer immunotherapies that harness RIPK1’s ability to trigger immunogenic cell death (Yatim et al., 2015).

## STAR★Methods

### Key Resources Table

**Table:** 

REAGENT or RESOURCE	SOURCE	IDENTIFIER
**Antibodies**		

Anti-RIPK1 (C-terminal)	BD Bioscience	610459
Anti-HOIL	Gift from Henning Walczak	N/A
Anti-cIAP1	Enzo Life Sciences	ALX-803-335-C100
Anti-TNFR1	Abcam	19139
Anti-Actin	Santa Cruz Biotechnology	sc-1615
Anti-p65	Cell Signaling	8242
Anti-P-p65	Cell Signaling	3033
Anti-IkB α	Santa Cruz Biotechnology	sc-371
Anti-P-p38	Cell Signaling	9215
Anti-p38	Cell Signaling	9212
Anti-P-JNK	Cell Signaling	9255
Anti-JNK	Santa Cruz Biotechnology	sc-571
Anti-P-ERK	Cell Signaling	9101
Anti-ERK	Gift from Chris Marshall	N/A
Anti-Caspase 8	Cell Signaling	9429
Anti-Ubiquitin	Dako	Z0458
Anti-cFLIP	Adipogene	AG-20B-0056
Anti-FADD	Santa Cruz Biotechnology	sc-6036
Anti-RIPK3	ProSci	2283
Anti-Tubulin	Sigma	T-9026
Anti-Sharpin	ProteinTech	14626-1-AP
Anti-TRAF2	Cell Signaling	4712
Anti-CD8-PE-Cy7	eBioscience	MHCD0812
Anti-GR-1-PE-Cy7	eBioscience	A14748
Anti-CD11c-FITC	eBioscience	11-0116-41
Anti-CD4-FITC	eBioscience	MCD0401
Anti-CD11b-Cy5	eBioscience	53-0112-82
Anti-B220-FITC	eBioscience	11-0460-82
Anti-CD69-PE	eBioscience	12-0691-82
Anti-CD3-APC	eBioscience	47-0032-82
Anti-CD16	eBioscience	14-0161-82

**Chemicals, Peptides, and Recombinant Proteins**		

Mouse recombinant TNF	Enzo Life Sciences	ALX-522-009-C050
OTULIN	In house	N/A
vOTU	In house	N/A
USP21	In house	N/A
PR619	2B Scientific	SI9619
GSK’963 (RIPK1 inhibitor)	Gift from GSK	N/A
GST-TUBE	In house	N/A
Glutathione Sepharose 4B	GE Healthcare	17-0756-01
cOmplete EDTA-free protease inhibitor tablets	Roche	11873580001
3xFLAG-hTNF	In house	N/A
Anti-FLAG M2 Affinity Gel	Sigma	A2220
1x FLAG-TNF	Enzo Life Sciences	ALX-804-034-C050
zVAD-FMK	Apex Bio	A1902
Protein G Sepharose	Sigma	P3296
Hoechst	Thermo Scientific	33342
Propidium iodide solution	Sigma	P4864
Various recombinant human *cIAP2* and *TRAF2* segments	In house	N/A
ULP1 protease	In house	N/A
Ac-DEVD-AMC	Sigma	A1086
3-AT	Formedium	3AT010
PreScission	GE Healthcare	27-0843-01
Compound A (Smac mimetic)	TetraLogic Pharmaceuticals	N/A
MG132	Sigma	C2211
Biotinylated SM	Gift from X. Wang	N/A
(5Z)-7-Oxozeaenol (TAK1 inhibitor)	Tocris Bioscience	3604
TWEAK	Enzo	522-021-c10

**Critical Commercial Assays**		

Duolink *In Situ* Detection Reagents Green	Sigma	DUO92014

**Deposited Data**		

Raw data	Mendeley	https://doi.org/10.17632/f559yfv4h4.1

**Experimental Models: Cell Lines**		

Primary MEFs WT, *cIAP2*^*−/−*^*cIAP1*^*−/−*^*, cIAP2*^*−/−*^*, cIAP2*^*−/−*^and *cIAP2*^*−/−*^*cIAP1*^*UBAmut*^	In house	N/A
Primary MDFs WT, *cIAP2*^*−/−*^ and *cIAP2*^*−/−*^*cIAP1*^*UBAmut*^	In house	N/A
Primary BMDMs WT, *cIAP2*^*−/−*^ and *cIAP2*^*−/−*^*cIAP1*^*UBAmut*^	In house	N/A
Primary Keratinocytes WT and *cIAP2*^*−/−*^*cIAP1*^*UBAmut*^	In house	N/A
Primary Splenocytes WT and *cIAP2*^*−/−*^*cIAP1*^*UBAmut*^	In house	N/A
Flp-In™T-REx™-HEK293	Thermo Scientific	R78007

**Experimental Models: Organisms/Strains**		

Mouse: C57BL/6 *cIAP2*^*−/−*^*cIAP1*^*loxP/loxP*^	Gift from John Silke	N/A
Mouse: C57BL/6 *cIAP2*^*FRT/FRT*^*cIAP1*^*minigene*^	In house	N/A
Mouse: C57BL/6 *cIAP2*^*−/−*^*cIAP1*^*minigene*^	In house	N/A
Mouse: C57BL/6 *cIAP2*^*−/−*^*cIAP1*^*UBAmut*^	In house	N/A

**Recombinant DNA**		

pTRIPZ	GE Dharmacon	RHS4696
pTRIPZ-*shcIAP1*	Open Biosystems	N/A
pCDNA5.5/FRT/TO vector	Thermo Fisher Scientific	V652020
pOG44	Thermo Fisher Scientific	V600520
pGBT9	Clontech/Takara	N/A
pACT2	Clontech/Takara	N/A
pGEX6P-1	GE Healthcare	N/A

**Software and Algorithms**		

Image Lab V5.2.1.	Bio-Rad laboratories	N/A
Origin	MicroCal	N/A
Swiss-Prot	https://www.ebi.ac.uk/uniprot	N/A
GraphPad Prism v6.0	https://www.graphpad.com/	N/A

### Contact for Reagent and Resource Sharing

Further information and requests for reagents may be directed to and will be fulfilled by the Lead Contact, Pascal Meier (pmeier@icr.ac.uk).

### Experimental Model and Subject Details

#### Mice generation

The cIAP2 and cIAP1 genes are positioned 10 Kb apart on the same chromosome. Hence, they recombine as a single genomic locus. The *cIAP2*^*FRT/FRT*^*cIAP1*^*minigene*^ mouse was generated by electroporating the *cIAP1* targeting vector into *C57BL6*-derived ES cells that were previously targeted on the *cIAP2* locus (Moulin et al., 2012). For the cIAP1 targeted allele the M396A and F398A mutations were introduced into exon 4 and a *F3* site-flanked *PGK-Hygro* selection cassette was inserted upstream of the genetically altered exon 4. A mini-gene corresponding to exon 4-7 of cIAP1 and a BGH poly-A signal were placed upstream of the selection cassette. The cDNA mini-gene is flanked by *loxP* sites. Mice carrying the *cIAP2*^*FRT/FRT*^*cIAP1*^*minigene*^ alleles were crossed to transgenic mice expressing the *FLPo* recombinase to generate the *cIAP2*^*−/−*^*cIAP1*^*minigene*^ animals. The *PGK-Hygro* resistance cassette on the cIAP1 targeted allele was also deleted by the FLPo-mediated recombination. The *cIAP2*^*−/−*^*cIAP1*^*minigene*^ mice were subsequently crossed to transgenic mice expressing the *Cre* recombinase to delete the mini-gene and generate the *cIAP2*^*−/−*^*cIAP1*^*UBAmut*^ animals. *cIAP2*^*−/−*^*cIAP1*^*loxP/loxP*^ mice were previously described (Moulin et al., 2012). Mice were kept according to the UK Home Office regulations. In vivo experiments were conducted according to institutional, national and European animal regulations. Animal protocols were approved by the Ethics Committee of Ghent University.

#### Mice injections monitoring and sampling

Experiments in mice were performed at the Department of Pharmacology of the Faculty of Veterinary Medicine of the Ghent University, Belgium, according to institutional, national and European regulations. Animal protocols were approved by the ethics committee of Ghent University. mTNF was diluted in endotoxin-free PBS and injected intravenously (i.v.) in a volume of 0.2 ml. Rectal body temperature was recorded with a digital thermometer (model 2001; Comark Electronics). Plasma samples and tissue samples of liver were collected at designated times after injection. Blood was obtained by cardiac puncture.

#### Cell lines

Primary and immortalized MEFs, primary MDFs and *Flp-In*^*TM*^*-Rex*^*TM*^*-HEK293* cells were cultured in Dulbecco’s modified Eagle’s Medium (DMEM) supplemented with 10% Fetal Bovine Serum (FBS), penicillin and streptomycin under 10% CO2.

### Method Details

#### Isolation of primary cells

Primary Mouse Embryonic Fibroblasts (MEFs) were generated from E13.5 embryos. After removing the placenta, yolk sac, head and the dark red organs, embryos were finely minced and digested for 20 min in 0.25% trypsin. Single cell suspension was then obtained by pipetting up and down the digested embryos. Mouse Dermal Fibroblasts (MDFs) were isolated as described in Etemadi et al. (2015). To generate Bone Marrow Derived Macrophages (BMDMs), bone marrow cells from tibia and femur of 2 month old mice were seeded in non-coated Petri dishes and cultured for 6 days in Dulbecco’s modified Eagle medium + 10% fetal bovine serum + 20% (v/v) L929 mouse fibroblast conditioned medium. Keratinocytes were isolated as described in Lichti et al. (2008). Splenocytes were isolated from 2 month old mice. Mouse spleens were mashed through a cell strainer into the Petri dish using the plunger end of a syringe. Cells were then washed once in cold PBS and treated with 1X Red Blood Cell Lysis Buffer (BioLegend, Cat N 420301) for 5 min on ice. Cells were then washed again in PBS and counted.

#### Splenocytes FACS analysis

5 × 10^5^ splenocytes were resuspended in 500 μL of cold PBS and stained with DAPI (1/5000) for 20 min on ice. Cells were then washed with cold PBS and resuspended in 50 μL Staining Buffer. 2 μL of blocking antibody (Anti-Mouse CD16/CD32) were added, and cells were kept on ice for 10 min. 50 μL of Staining Buffer containing the desired antibodies were then added and cells were kept on ice for 30 min. Cells were washed in cold PBS, resuspended in 1 mL of cold PBS and analyzed by FACS.

#### Cell culture, constructs and transfection

For the generation of the 293 stable cell lines, where endogenous cIAP1 was reconstituted with WT and mutant cIAP1, *Flp-InTM T-RExTM HEK293*^*shcIAP*1^ cells were created. First *Flp-In*^*TM*^*T-REx*^*TM*^*-HEK293* cells (Invitrogen) were transduced with lentiviral particles targeting the 3′ UTR of the *cIAP1* mRNA. To this end, we used *pTRIPZ-shcIAP1* (Open Biosystems), which allows Doxocycline-inducible expression of a miR30-based *shcIAP1* RNA. After puromycin selection, individual clones were tested for *cIAP1* knockdown efficiency. Such cells were further tested functionally using TNF signaling and NIK activation as readouts. Next, the respective *cIAP1* constructs were cloned into *pcDNA5.1/FRT/TO* (Invitrogen). The empty *pcDNA5.1/FRT/TO-2HA-Strep* plasmid was used as a control. To generate site-specific single copy insertions, *pcDNA5.1FRT/TO*-based plasmids were co-transfected with *pOG44* into *Flp-In*^*TM*^*T-REx*^*TM*^*-HEK293*^*shcIAP1*^ cells. After selection with hygromycin, stable cells were selected. Endogenous cIAP1 suppression and expression of WT or mutant versions of cIAP1 were simultaneously induced by treating cells with 100 ng/ml Doxycycline for at least 48 hours.

#### Reagents, Constructs and Antibodies

The GSK’963 RIPK1 kinase inhibitor was provided by GSK. The following antibodies were used: α-RIPK1 (BD Biosciences, 610459), α-HOIL (gift from Henning Walczak), α-cIAP1 (Enzo, ALX-803-335-C100), α-TNFR1 (Abcam, 19139), α-Actin (Santa Cruz Biotechnology, sc-1615), α-P-p65 (Cell Signaling, 3033), α-p65 (Cell Signaling, 8242), α-IkBα (Santa Cruz, sc-371), α-P-p38 (Cell Signaling, 9215), α-p38 (Cell Signaling, 9212), α-P-JNK (Cell Signaling, 9255), α-JNK (Santa Cruz Biotechnology, sc-571), α-P-ERK (Cell Signaling, 9101), α-ERK (gift from Chris Marshall) α-caspase-8 (Cell Signaling, 9429), α-FLAG [M2] (SIGMA, M8823), α-Ub (Dako, Z0458), α-FLIP (Adipogene, AG-20B-0056), α-FADD (Santa Cruz Biotechnology, sc-6036), α-RIPK3 (ProSci, 2283), α-Tubulin (SIGMA, T9026), α-SHARPIN (Proteintech, 14626-1-AP), α-TRAF2 (Cell Signaling, 4712), α-CD8-PE-Cy7, GR-1-PE-Cy7, CD11c-FITC, CD4-FITC, CD11b-Cy5, B220-FITC (gift from Henning Walczak), α-CD69-PE (eBioscience, 12-0691-82), α-CD3-APC (eBioscience, 47-0032-82), and α-CD16 (eBioscience, 14-0161-82).

#### UbiCRest analysis

The UbiCRest analysis with linkage selective DUBs was performed essentially as described in Hospenthal et al. (2015). Briefly, the release fraction (see complex-I purification) was incubated with the following DUBs: 1 μM OTULIN, 0.5 μM vOTU, 1.5 μM USP21. The reaction was conducted in the presence of 1 mM DTT for 30 min at 37°C. Reactions were stopped with SDS sample buffer, and the ubiquitylation status analyzed by western blotting.

#### Tube Assay

Cells were lysed in DISC lysis buffer (20 mM Tris-HCL pH7.5, 150 mM NaCl, 2 mM EDTA, 1% Triton X-100, 10% glycerol) supplemented with protease inhibitors, 1 mM DTT, PR619 (10 μM) and GST-TUBE (50 μg/ml; 50 μg TUBE/mg protein lysate). Cell lysates were rotated at 4°C for 20 min then clarified at 4°C at 14,000 rpm for 10 min. 20 μL GST beads were added and immunoprecipitations were performed overnight. Beads were washed 4x in wash buffer (50 mM Tris pH 7.5, 150 mM NaCl, 0.1% Triton X-100, and 5% glycerol) + PR619 (10 μM), and bound proteins eluted by boiling in 50 μ l 1x SDS loading dye.

#### Complex-I/II Purification

Cells were seeded in 15 cm dishes and treated as indicated with 3x FLAG-hTNF (5 μg/ml). To terminate stimulation, media was removed and plates were washed with 50 mL of ice cold PBS. Plates were frozen at −80°C until all time points were acquired. Plates were thawed on ice and cells were lysed in 1% Triton X-100 lysis buffer (30 mM Tris-HCl pH 7.4, 120 mM NaCl, 2 mM EDTA, 2 mM KCl, 10% glycerol and 1% Triton X-100, supplemented with protease inhibitors and PR619 (10 μM). Cell lysates were rotated at 4°C for 20 mins then clarified at 4°C at 14,000 rpm for 30 mins. Proteins were immunoprecipitated from cleared protein lysates with 20 μL of α-FLAG M2 beads (SIGMA) with rotation overnight at 4°C. For the 0 hr sample 5 μg/ml of FLAG-TNF were added post-lysis. 4x washes in 1% Triton X-100 buffer with PR619 (10 μM) were performed, and samples eluted by boiling in 60 μL 1x SDS loading dye. For complex-II purification cells were seeded in 10 cm dishes and treated as indicated using media containing 1x FLAG-TNF (100 ng/ml) and zVAD (10 μM). Cells were lysed on ice in 1% Triton X-100 lysis buffer (30 mM Tris-HCL pH 7.4, 120 mM NaCl, 2 mM EDTA, 2 mM KCl, 1% Triton X-100 supplemented with protease inhibitors and 10 μM PR619). Cell lysates were rotated at 4°C for 20 mins then clarified at 4°C at 14,000 rpm for 10 mins. 20 μL of protein G Sepharose (SIGMA), blocked for 1 hr with lysis buffer containing 1% BSA, were bound with FADD antibody (1.5 μg antibody/mg protein lysate) and were rotated with cleared protein lysates 4 hr at 4°C. 4x washes in lysis buffer were performed, and samples eluted by boiling in 80 μL 1x SDS sample buffer.

#### Cell death analysis

2 × 10^5^ cells (MEFs and MDFs) were seeded in six well plates and 24 hr later they were treated as indicated for an additional 24 hr. Hoechst 33342 (10 μg/ml) and Propidium Iodide (PI) (1 μg/ml) were added. After 2 mins 10 to 15 images per well were taken with a fluorescent inverted microscope and the ration of dead/live cells were counted manually. 5 × 10^4^ BMDMs were seeded in 96 well plates and 24 hr later they were treated as indicated for an additional 24 hr. Hoechst (0.5 μg/ml) and PI (1 μg/ml) were added and the ratio dead/live cells was measured using the Celigo imaging system.

#### Production of recombinant proteins and isothermal titration calorimetry

Various human *cIAP2* and *TRAF2* segments were cloned into *pSUMO* vector respectively to produce N-terminally His-SUMO tagged proteins. The constructs were then transformed into BL21 (DE3) cells and cultured in LB medium at 37°C, respectively. Protein expression was induced overnight at 20°C with 0.5 mM IPTG when OD_600_ reached 0.8. Cells were lysed in buffer containing 25 mM Tris-HCl at pH 8.0, 150 mM NaCl, 10 mM imidazole and 5 mM β-mercaptoethanol. The recombinant proteins were affinity-purified by Ni-Sepharose beads (GE Healthcare Life Sciences). The SUMO tag was removed by overnight digestion with homemade ULP1 protease at 4°C. The untagged proteins were further purified by HiTrap Q anion exchange and Superdex 200 gel filtration chromatography (GE Healthcare Life Sciences). Isothermal titration calorimetry measurements were performed at 16°C, using a MicroCal ITC_200_ microcalorimeter (MicroCal). For the TRAF2:cIAP2 interactions, the calorimetric titrations were performed by injecting 2 μL of cIAP2 protein solution (2–4 mM) into a sample cell containing 200 μl 0.20 mM TRAF2 protein in 25 mM Tris-HCl at pH 8.0, 150 mM NaCl. A total of 20 injections were performed with a spacing of 150 s and a reference power of 6 μcal/s. Binding isotherms were plotted and analyzed using Origin Software (MicroCal).

#### Caspase activity assay (DEVDase)

2 × 10^5^ cells (MEFs) were plated in 6-well plates and treated as indicated in 2 mL for the indicated times. After treatment, media was removed, and 300 μL 1% DISC lysis buffer (20 mM Tris-HCL pH 7.5, 150 mM NaCl, 2 mM EDTA, 1% Triton X-100, 10% glycerol) was added to each well, cells were scraped and lysates were left on ice for 5 min. 50 μL of lysate per condition were transferred into a 24 well plate and 450 μL DEVDase assay mix (20 mM Ac-DEVD-AMC (Sigma), 1 mM DTT, 50 mM Tris pH 7.5, 150 mM NaCl, 0.1% Triton X-100, and 5% glycerol) was added to each well (NB: cell lysates were not cleared). Plates were wrapped in foil and reactions allowed to proceed by incubation at room temperature for up to 24 hr. DEVDase activity was read at 380 nM excitation/460 nM emission.

#### Yeast two- and three-hybrid

The yeast strain Y2HGold (Clontech) was co-transformed with the respective bait and prey plasmids. Positive transformants were selected on minimal SD-Leu-Trp medium (Formedium). Three single colonies for each bait and prey co-transformation were patched out on fresh SD-Leu-Trp plates and grown for 2 days at 30°C. Each patch was resuspended in 180 μL of sterile water in a 96-well plate and replica plated onto non-selective (SD-Leu-Trp) or selective medium (SD-Leu-Trp-His), containing the indicated concentration of 3-amino-1,2,4-triazole (3-AT, Formedium). Yeast plates were incubated at 30°C for 1 week. UbcH5b prey vector was provided by Rachel Klevit.

#### Protein expression and purification

BL21 cells were transformed with pGEX6p-1-3XFLAG-TNF plasmid. One colony was picked and incubated o/n in 100 mL LB medium with 100 μg/ml ampicillin. Next day 900 mL of LB medium without AMP were added, cells were grown for 1 h at 37°C and then 0.5 mM IPTG was added for further 4 h. Bacteria were spun for 15 min at 4000 rpm, the supernatant was discarded and the pellet was lysed in 10 mL Triton X-100 lysis buffer (10 mM Tris pH 7.5, 150 mM NaCl, 10% glycerol and 1% Triton X-100 with complete protease inhibitor cocktail). The lysate was sonicated and left for 15 min at 4°C and clarified for 30 min at 140,000 rpm at 4°C. Lysate was rotated with glutathione Sepharose beads for 4 hr at 4°C and 3x washed with IPPG150 buffer (0,1% Triton X-100, 50 mM Tris pH 7.5, 150 NaCl, 5% glycerol) were performed, followed by a final wash in PreScission cleavage buffer (50 mM Tris pH 7, 150 mM NaCl, 1 mM EDTA and 1 mM DTT). For PreScission cleavage 500 μL cleavage buffer and 30 μL PreScission enzyme were added to the beads at 4°C o/n. The beads were spun, and supernatants were collected and passed through Thermo Scientific buffer exchange columns to remove bead contamination. Recombinant TNF concentration was determined on Coomassie Blue-stained polyacrylamide gel using known amount of BSA as standard, and quantitated using Image Lab software.

#### Proximity ligation assay (PLA)

PLA was performed according to the manufacturer’s protocol using the Duolink Detection Kit (SIGMA). Cells were examined with a confocal microscope (Objective 40x, Zeiss LSM 710).

#### Ub Chain Composition Mass Spectrometry Analysis

Ub chains were separated on a NuPAGE 4%–12% gradient gel (Invitrogen) before in-gel digestion with trypsin and the addition of Ub-AQUA peptide internal standards according to Kirkpatrick et al. (2006). 10 μL of each sample was directly injected onto an EASY-Spray reverse-phase column (C18, 3 μm, 100 Å, 75 μm × 15 cm) using a Dionex UltiMate 3000 high-pressure liquid chromatography system (Thermo Fisher Scientific) and analyzed on a Q-Exactive mass spectrometer (Thermo Fisher Scientific) using parallel reaction monitoring (PRM), similar to Tsuchiya et al. (2013). Data were analyzed further according to Kirkpatrick et al. (2006)

### Data and Software Availability

Raw data have been deposited to Mendeley Data and are available at https://doi.org/10.17632/f559yfv4h4.1.

## Supplemental Information

Document S1. Figures S1–S5

Document S2. Article plus Supplemental Information
