# Supplementary material for: Ubiquitin-Mediated Regulation of RIPK1 Kinase Activity Independent of IKK and MK2
Source: Mol Cell. 2018 Feb 15;69(4):566–580.e5. doi: 10.1016/j.molcel.2018.01.027 (PMC5823975; doi:10.1016/j.molcel.2018.01.027)
Supplement: Document S1. Figures S1–S5 [file mmc1.pdf]

Molecular Cell, Volume *69*

Supplemental Information

Ubiquitin-Mediated Regulation of RIPK1 Kinase Activity Independent of IKK and MK2

Alessandro Annibaldi, Sidonie Wicky John, Tom Vanden Berghe, Kirby N. Swatek, Jianbin Ruan, Gianmaria Liccardi, Katiuscia Bianchi, Paul R. Elliott, Sze Men Choi, Samya Van Coillie, John Bertin, Hao Wu, David Komander, Peter Vandenabeele, John Silke, and Pascal Meier

Supplementary Information

Figure S1

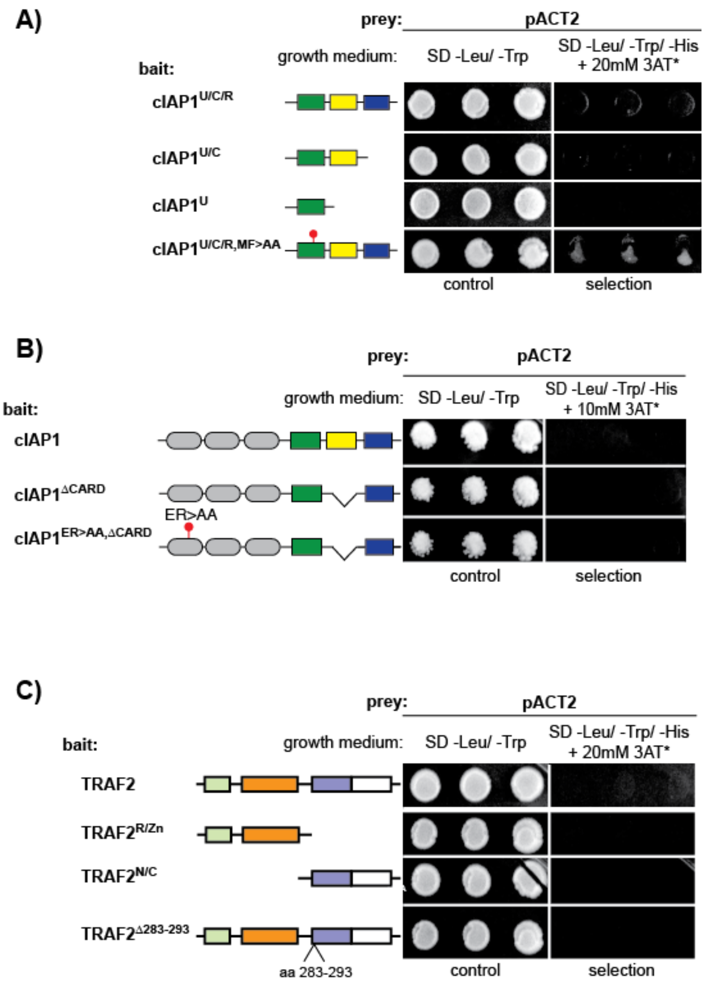

Supplementary Figure S1. Interaction of the UBA domain of cIAP1 with TRAF2, Related to Figure 1

(A-C) Yeast two-hybrid negative control. The indicated bait constructs were co-transformed with the empty prey vector pACT2 to rule out autonomous growth.

Figure S2

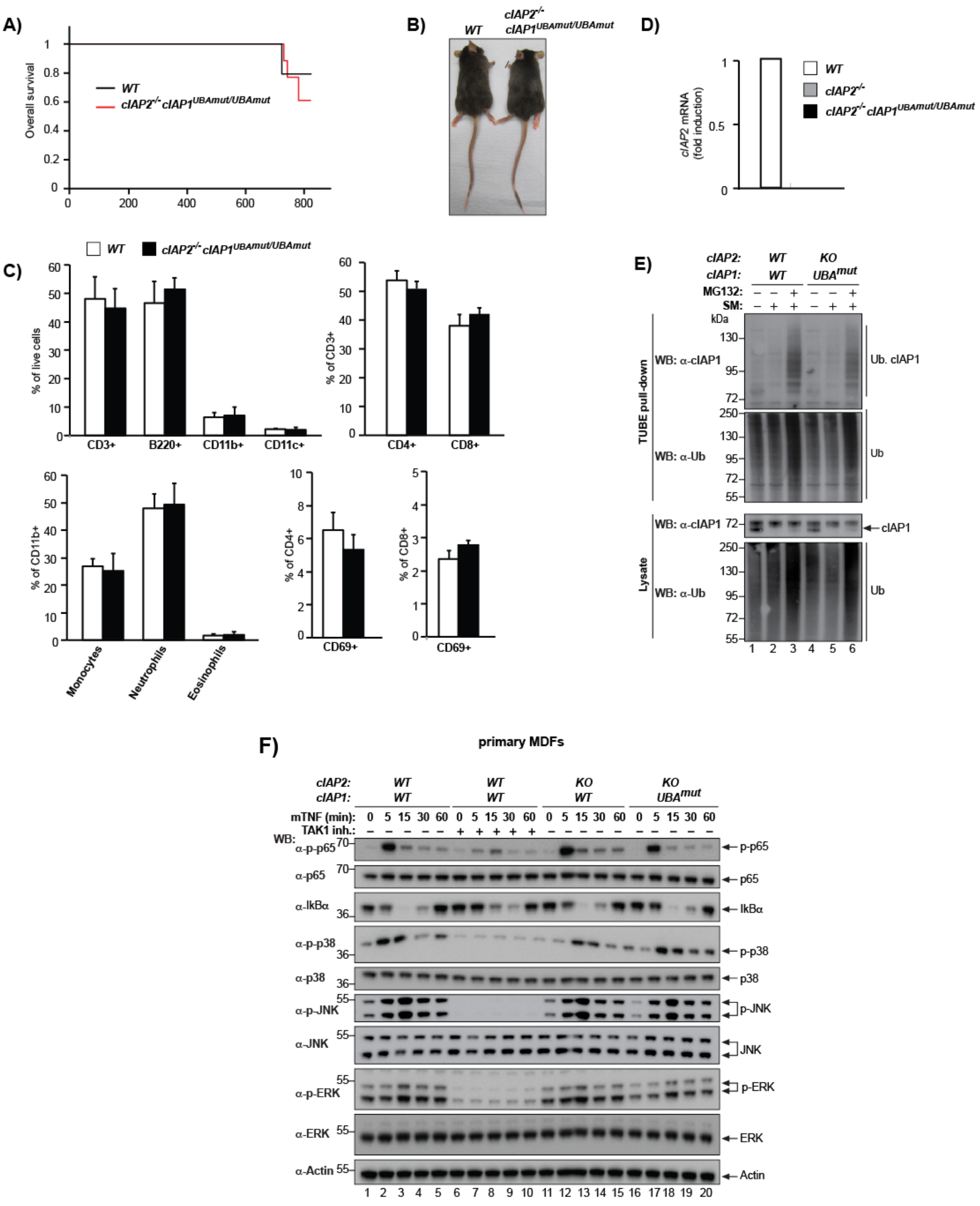

Supplementary Figure S2. The UBA domain of cIAP1 is dispensable for normal development and NF-KB-induced gene expression, Related to Figure 3

(A) Aging curves for *WT* and *cIAP1^{UBAmut}* mice. **(B)** Representative images of 2 months old *WT* and *cIAP1^{UBAmut}* mice. **(C)** FACS analysis of hematopoietic cells isolated from the spleen of *WT* and *cIAP1^{UBAmut}* mice. Cells were analysed using antibodies for the indicated cell surface markers. **(D)** RT-

PCR analysis of *cIAP2* mRNA levels in *WT*, *cIAP2$^{-/-}$* and *cIAP1^{UBAmut}* MEFs. **(E)** TUBE affinity purification of the ubiquitylated proteome from *WT* and *cIAP1^{UBAmut}* MEFs treated with the indicated agents. TUBE pull-down was followed by Western blot analysis with the indicated antibodies. **(F)** Western blot analysis of MDFs with the indicated genotypes treated with TNF and harvested at the indicated times points.

Figure S3

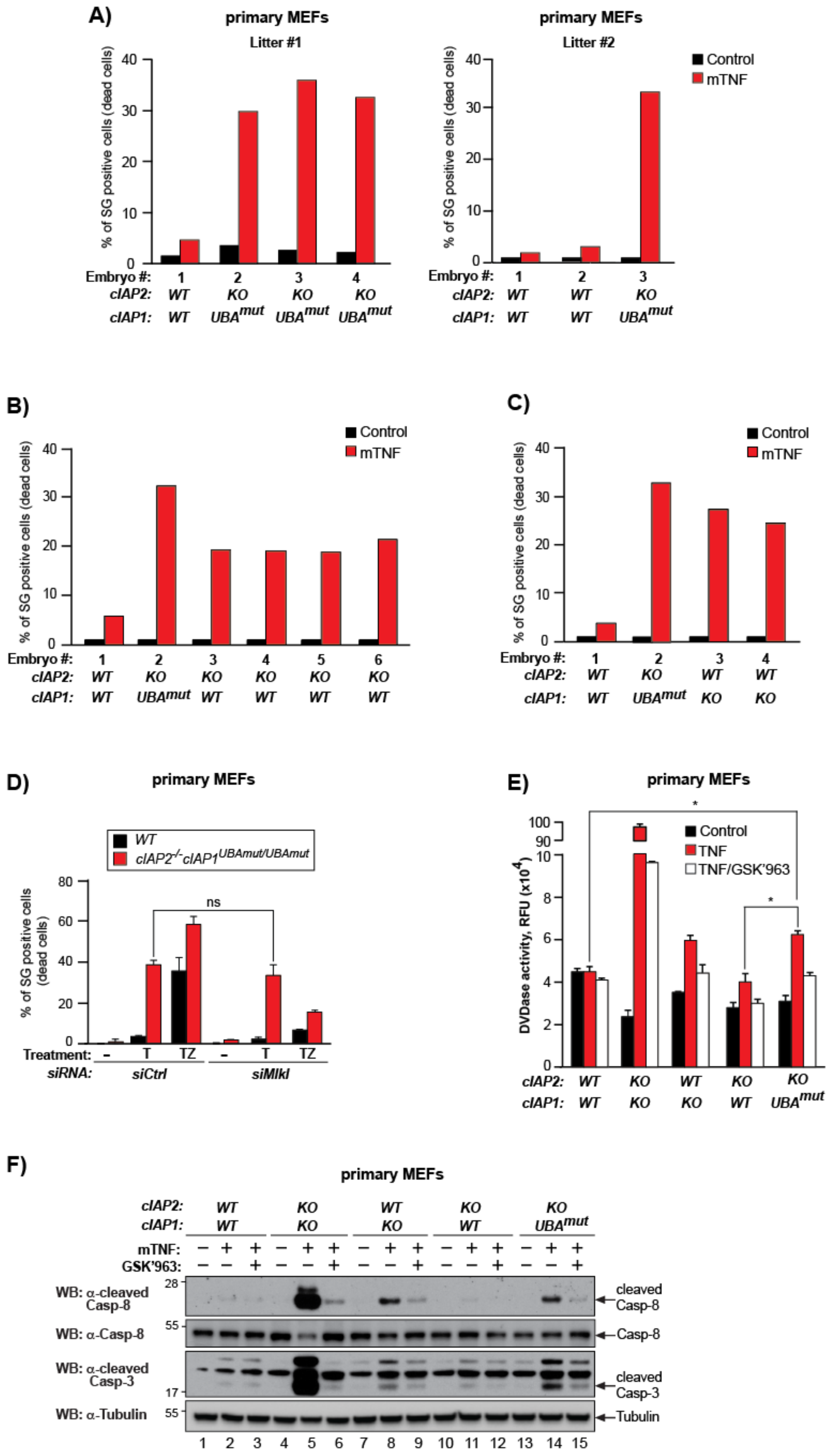

Supplementary Figure S3. Mutation in the UBA domain switches the TNF response to cell death, Related to Figure 5

(A-C) Primary MEFs of the indicated genotypes were treated with TNF (100 ng/ml) for 24 hrs followed by quantification of PI positive cells. **(D)** *WT* and *cIAP1^{UBAmut}* primary MEFs were transfected either with siRNA control or with siRNAs targeting *Mlkl*. After 72 hrs following transfection, cells were treated as indicated, and cell death was measured by scoring PI positive cells. **(E)** DVDase analysis of primary MEFs of the indicated genotypes subjected to indicated treatments for 8 hrs (z-VAD-FMK 10 μM), data are presented as mean ± SD, n=3, * $p<0.05$, statistics were performed using two-way ANOVA. **(F)** Western blot analysis of activated caspase-8 and caspase-3 of the indicated MEFs treated with the indicated agents for 12 hrs.

Figure S4

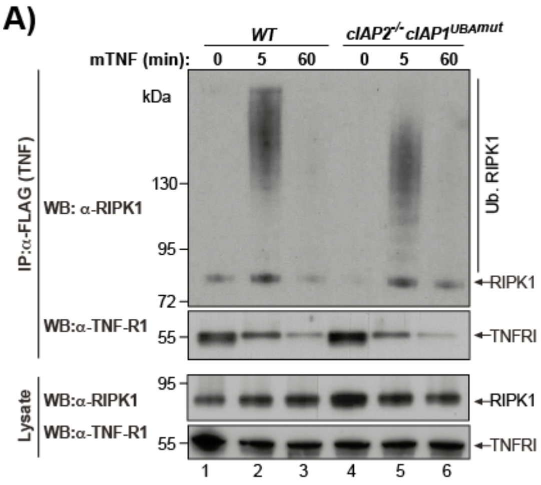

Supplementary Figure S4. The UBA domain facilitates cIAP1-mediated degradation of RIPK1, Related to Figure 6

(A) Purification of the TNF-receptor signalling complex (complex-I) from primary MEFs of the indicated genotypes. Cells of the indicated genotypes were treated with FLAG-TNF for 0, 5 and 60 mins. Cell lysates were then subjected to FLAG immune-precipitation followed by Western blot analysis with the indicated antibodies. Representative images of at least three independent experiments are shown.

Figure S5

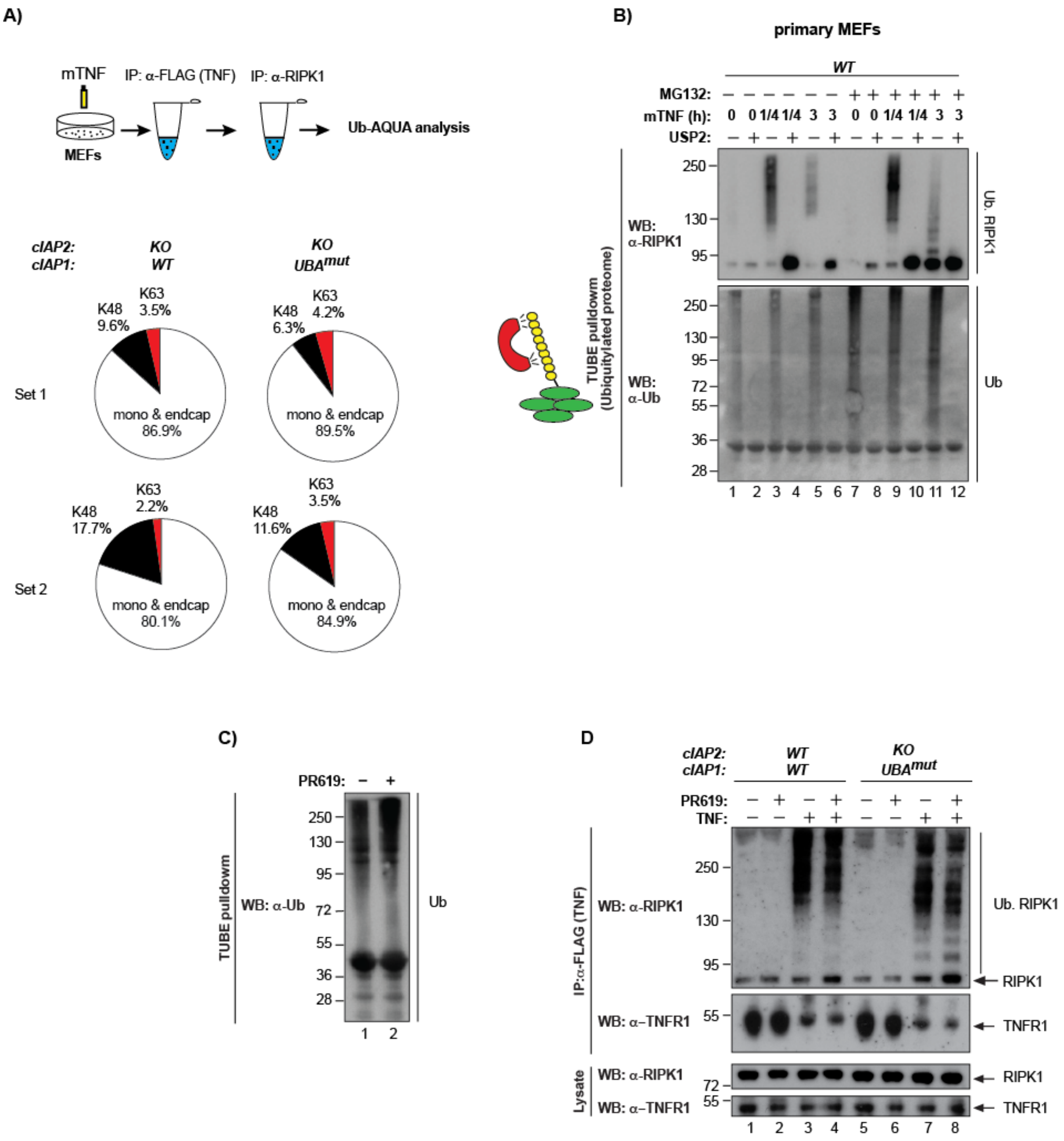

Supplementary Figure S5. cIAP1 targets RIPK1 to proteasomal degradation, Related to Figure 7

(A) Absolute quantification (AQUA)-based mass spectrometry of Ub chain linkage types on complex-I derived RIPK1 from *cIAP2*$^{-/-}$ and *cIAP1*^{UBAmut} MEFs (two independent replicates). Pie charts indicate the ubiquitin linkage types and mono- and endcap-Ub. Values were normalized to the total amount of Ub. **(B)** *WT* MEFs, either pre-incubated with MG132 for 1 hour or left untreated, were treated with TNF (100 ng/Ml) for the indicated time points. Cell lysates were subjected to TUBE pull-down followed by USP2 digestion. Western blot analysis for the indicated proteins was then carried out. **(C)** WT MEFs were treated with the pan-DUB inhibitor PR619 for 2 hrs. Cell lysates were subjected to TUBE pulldown followed by Western blot analysis using an Ubiquitin specific antibody. **(D)** WT MEFs were

treated with TNF in the presence of the pan-DUB inhibitor PR619 for 2 hrs, and complex-I was purified using FLAG-TNF as affinity reagent. The presence of the indicated proteins was analysed using the indicated antibodies.
